# Nitrogen Fixation and Hydrogen Evolution by Sterically Encumbered Mo-Nitrogenase

**DOI:** 10.1021/jacsau.3c00165

**Published:** 2023-05-09

**Authors:** Cécile Cadoux, Daniel Ratcliff, Nevena Maslać, Wenyu Gu, Ioannis Tsakoumagkos, Sascha Hoogendoorn, Tristan Wagner, Ross D. Milton

**Affiliations:** † Department of Inorganic and Analytical Chemistry, Faculty of Sciences, University of Geneva, Quai Ernest-Ansermet 30, 1211 Geneva 4, Switzerland; ‡ National Centre of Competence in Research (NCCR) Catalysis, University of Geneva, Quai Ernest-Ansermet 30, 1211 Geneva 4, Switzerland; § Max Planck Institute for Marine Microbiology, Celsiusstraße 1, 28359 Bremen, Germany; ∥ Department of Chemical Engineering, Stanford University, Stanford, California 94305, United States; ⊥ Department of Organic Chemistry, National Center of Competence in Research (NCCR) Chemical Biology, University of Geneva, Quai Ernest-Ansermet 30, 1211 Geneva 4, Switzerland

**Keywords:** nitrogenase, cooperativity, nitrogen fixation, ammonia, hydrogen, metalloenzyme

## Abstract

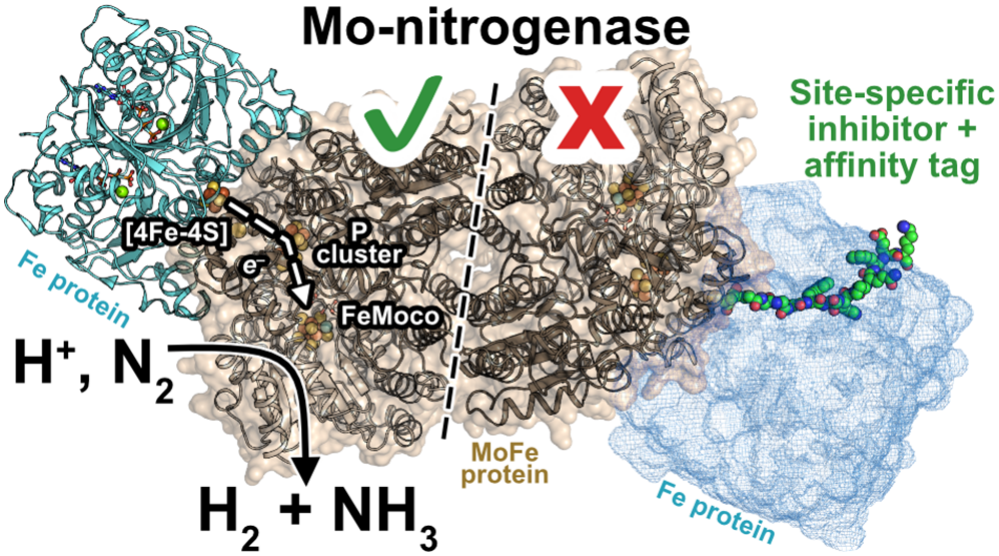

The substrate-reducing proteins of all nitrogenases (MoFe, VFe, and FeFe) are organized as α_2_ß_2_(γ_2_) multimers with two functional halves. While their dimeric organization could afford improved structural stability of nitrogenases *in vivo*, previous research has proposed both negative and positive cooperativity contributions with respect to enzymatic activity. Here, a 1.4 kDa peptide was covalently introduced in the proximity of the P cluster, corresponding to the Fe protein docking position. The Strep-tag carried by the added peptide simultaneously sterically inhibits electron delivery to the MoFe protein and allows the isolation of partially inhibited MoFe proteins (where the half-inhibited MoFe protein was targeted). We confirm that the partially functional MoFe protein retains its ability to reduce N_2_ to NH_3_, with no significant difference in selectivity over obligatory/parasitic H_2_ formation. Our experiment concludes that wild-type nitrogenase exhibits negative cooperativity during the steady state regarding H_2_ and NH_3_ formation (under Ar or N_2_), with one-half of the MoFe protein inhibiting turnover in the second half. This emphasizes the presence and importance of long-range (>95 Å) protein–protein communication in biological N_2_ fixation in *Azotobacter vinelandii*.

## Introduction

**Figure 1:**
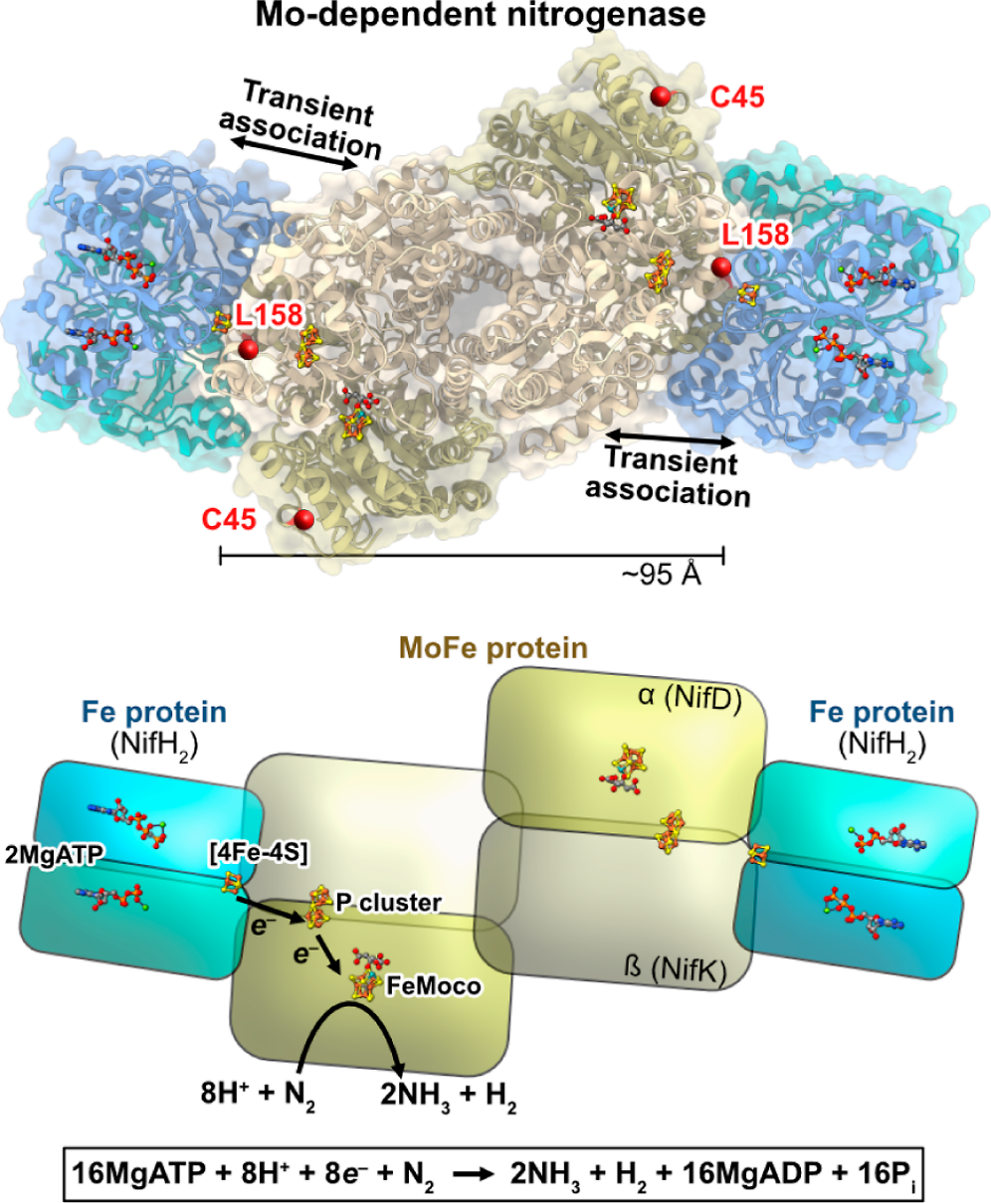
Structure of the Mo-dependent nitrogenase transition-state complex between the Fe protein and MoFe protein (PDB: 4WZA). In this complex, 2MgATP was replaced with MgADP and non-hydrolyzable MgAMPPCP to enable stable complex formation. The α-C45 and α-L158 residues are highlighted with red spheres. Fe = rust, S = yellow, Mo = cyan, C = gray, O = red, N = blue, Mg = green, and P = orange.

The fixation of kinetically inert atmospheric dinitrogen (N_2_) to ammonia (NH_3_) is catalyzed in some specific microbes by a single family of enzymes known as nitrogenases, with turnover frequencies of around one N_2_ fixed per second and a second-order rate constant of ∼10^4^ M^–1^ s^–1^ (*k*_cat_/*K*_M_).^1−6^ The Mo-dependent nitrogenase consists of an N_2_-reducing molybdenum-iron (MoFe) protein and a corresponding reductase called the iron (Fe) protein (Figure 1). The MoFe protein is a ∼230 kDa (αß)_2_ heterotetramer (NifDK), where each αß half contains an electron-transferring [8Fe–7S] P cluster and a [7Fe–9S–C–Mo]/homocitrate FeMo cofactor (FeMoco).^2^ The Fe protein is a homodimeric NifH_2_ protein of ∼66 kDa containing a single [4Fe–4S] cluster and two MgATP binding sites. During turnover, each αß half of the MoFe protein accepts electrons from the ATP-hydrolyzing Fe protein, which are transferred via the P cluster to the FeMoco for N_2_ fixation to NH_3_ (eq 1)^4^
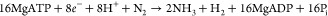
1

It is important to note that the above equation represents the optimized stoichiometry of one H_2_ produced per N_2_ fixed by the Lowe-Thorneley mechanism, with additional unproductive H_2_ formation taking place under non-optimal turnover.^1,7−10^ Each transient association of the Fe protein (Fe protein cycle) ultimately results in the transfer of 1*e*^–^ to the FeMoco, where each Fe protein cycle consists of at least (i) MgATP-bound Fe:MoFe association, (ii) electron transfer (ET) from the P cluster to the FeMoco, (iii) ET from the Fe protein’s [4Fe–4S] cluster to the P cluster, (iv) 2ATP hydrolysis, (v) the release of two inorganic phosphate (P_i_), and (vi) MgADP-bound Fe:MoFe dissociation. The rate-limiting step of nitrogenase catalysis is thought to be the release of P_i_ by the Fe protein, taking place with a rate constant of 25–27 s^–1^.^11^ This, in turn, implies that each Fe protein electron delivery cycle takes place with an overall rate constant of ∼13 s^–1^.

Importantly, it has been shown that the two αß halves of the MoFe protein do not function independently during their repeated transient associations with the Fe protein (for electron delivery), suggesting that communication between the Fe proteins ∼100 Å apart takes place during turnover.^12−14^ In 2016, Danyal et al. reported that the Fe protein electron delivery cycles experience negative cooperativity in the pre-steady-state.^12^ In other words, the Fe protein electron delivery cycle on one αß half suppresses ET in the other half. This was observed by quantitative measurements of Fe protein oxidation, ATP hydrolysis, and P_i_ release.^12^ In 2021, Truscott et al. reported positive cooperativity in the steady state for the reduction of acetylene, a non-physiological yet historically prominent substrate of nitrogenase.^13^ In this approach, cooperativity was investigated by forming inactive Fe:MoFe complexes on one αß half of the MoFe protein (using AlF_4_^–^ or nonstandard, tightly associating Fe proteins). Notably, cooperativity was not observed for 2H^+^ reduction to H_2_, a physiologically relevant reaction that is catalyzed by nitrogenase in both the absence and presence of N_2_. In 2011, Eady and co-workers also observed that the MoFe protein containing only one FeMoco (half-populated) undergoes additional, non-electron-transferring interactions with the half-reactive MoFe protein, although the absence of the FeMoco was previously found to introduce a large change in the conformation of the MoFe protein.^15,16^ More recently, cryogenic electron microscopic investigation into Fe–MoFe interactions during turnover identified a potential preference for the MoFe protein to associate to one Fe protein at a time, refocusing the spotlight on the MoFe protein’s arrangement as a heterotetramer with two functional αß halves.^17^

An important open question is therefore: how does cooperativity (negative, positive, or indeed none) impact N_2_ fixation by nitrogenase during continued turnover? Harris et al. recently proposed that decreased selectivity toward N_2_ fixation in the alternative vanadium-dependent and iron-only nitrogenases is due to decreased rate constants for the reductive elimination of H_2_ (an activational step for N_2_ fixation).^18^ This reductive elimination requires the delivery of at least 4*e*^–^ to the FeMoco for sufficient activation, which in turn requires ATP-hydrolysis-coupled electron transfer from the Fe protein in one αß half of the MoFe protein. Stalled electron transfer to the FeMoco (by, for example, negative cooperativity induced by the second αß half of the MoFe protein) then provides time for the non-productive evolution of H_2_ (and the loss of reducing equivalents) by the protonolysis of metal-hydrides on FeMoco.^10^ This competition between reductive elimination and metal-hydride protonolysis explains the “optimal” stoichiometry of one H_2_ evolved per N_2_ fixed.^7^ We hypothesized that the liberation of inhibited electron delivery to one αß half of the MoFe protein could therefore yield the stoichiometrically optimized production of one H_2_ per N_2_ fixed during continuous nitrogenase turnover, provided that cooperativity is not strictly necessary for N_2_ fixation. However, in order to observe this, we deemed it of utmost importance to study the half-reactivity on a MoFe protein that (i) contained FeMoco in both αß halves (retaining its native conformation^16^) and (ii) was not half-inhibited by a tightly associating Fe protein on one αß half, given that conformational changes transmitted between the Fe proteins bound to both αß halves are considered essential to cooperativity.^13,14^

Here, we report on the reactivity of a MoFe protein (from *Azotobacter vinelandii*) wherein we sought to selectively inhibit Fe protein association on only one αß half by steric inhibition. To achieve this, we employed a MoFe protein mutant possessing a single solvent-exposed Cys residue in proximity to the P cluster (α-C45A/L158C, NifD = α); this mutant was previously employed to conjugate a Ru-based photosensitizer in the place of the Fe protein, enabling photo-excited electron transfer to the P cluster.^19^ X-ray crystallography confirms the solvent accessibility (albeit somewhat geometrically hidden) of this cysteine residue. We subsequently employed the reactivity of this cysteine in a thiol-maleimide Michael addition to introduce a large synthetic Strep-tag-containing peptide (1.38 kDa) to facilitate both (i) Fe protein steric inhibition and (ii) the separation of inhibited MoFe proteins from the unmodified MoFe protein. This population of partially inhibited MoFe protein (lacking the uninhibited MoFe protein) was confirmed to be active for N_2_ fixation, where a maximum velocity (*V*_max_) of 66% was determined, consistent with negative cooperativity during N_2_ fixation. Importantly, we observed that the selectivity (product/electron distribution) of this partially inhibited MoFe was practically unchanged between 0 and 1 atm N_2_, suggesting that cooperativity may not contribute toward nitrogenase’s remarkable selectivity for N_2_. We conclude that negative cooperativity, globally, is employed by this nitrogenase for both H_2_ production and N_2_ fixation.

## Results and Discussion

### Structure of the α-C45A/L158C MoFe Protein

As shown in Figure 1, residue α-L158 is located at the Fe:MoFe protein interface, and we therefore hypothesized that the functionalization of a Cys residue in this location with a steric inhibitor could prevent access of the Fe protein (and, therefore, nitrogenase catalysis). Indeed, a nitrogenase α-C45A/L158C MoFe mutant has been previously reported, yielding a single solvent-exposed Cys in proximity to the P cluster.^19^ We first prepared this α-C45A/L158C MoFe with an N-terminal 8xHis tag on NifD (α subunit) for affinity purification, using a *sacB*-based markerless mutagenesis approach (Figure S1, Supporting Information).^20−22^

**Figure 2:**
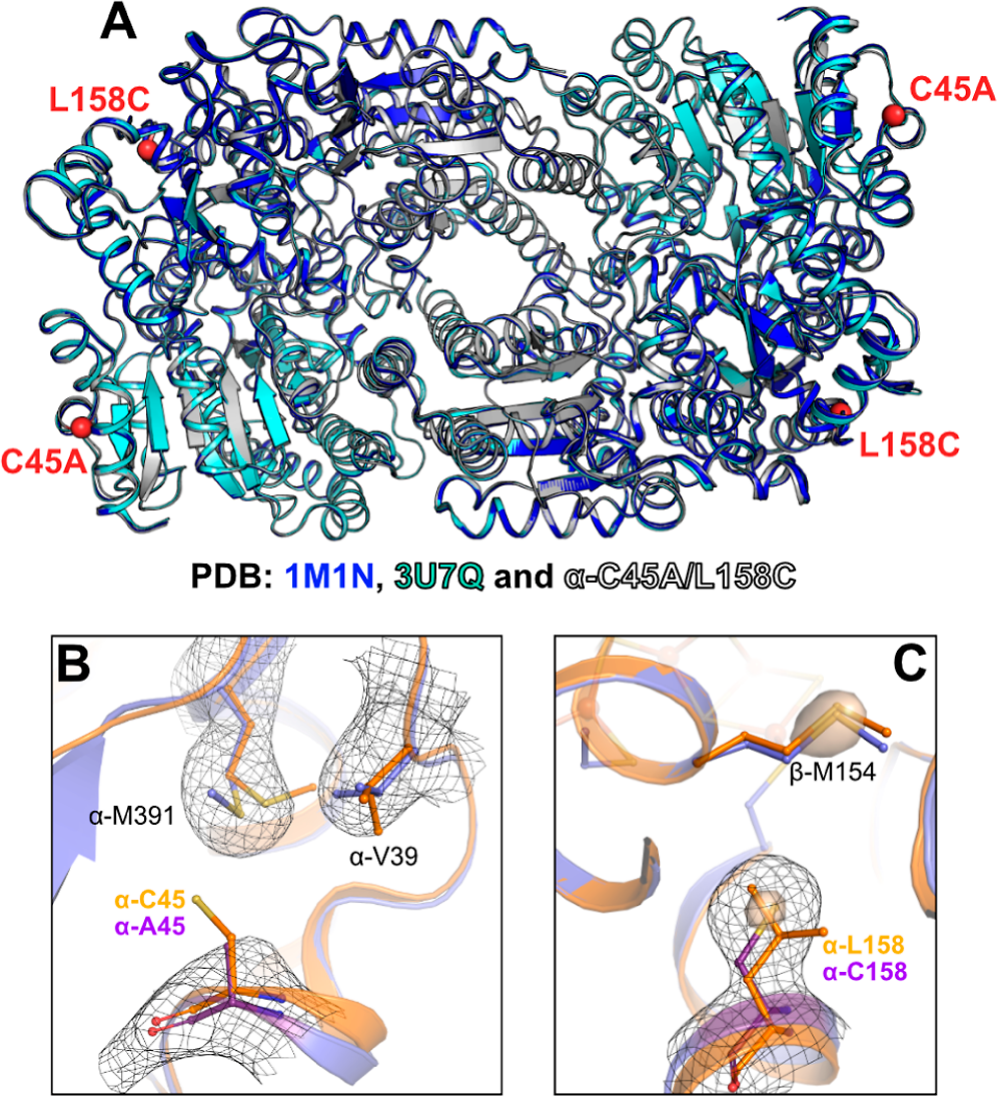
(A) X-ray crystal structure overlay of *A. vinelandii* MoFe proteins PDB 1M1N (1.16 Å resolution, blue), PDB 3U7Q (1.00 Å resolution, cyan), and the α-C45A/L158C MoFe protein from this work (3.03 Å resolution, white, PDB 8BTS). Electron densities of the (B) α-A45 (violet) and (C) α-C158 regions, overlaid with the X-ray crystal structure of the wild-type MoFe protein (PDB 3U7Q). The P cluster and FeMoco were omitted for clarity (Supporting Information). The 2*F*_o_–*F*_c_ electron density maps, contoured at 1.0 σ, are shown by a black mesh. In (C), an additional 2*F*_o_–*F*_c_ electron density contoured at 4.5 σ was superposed and represented as a transparent surface.

After verifying the introductions of the mutations by DNA sequencing (Supporting Information), we next crystallized the purified mutant MoFe protein and elucidated its structure by X-ray crystallography (Figure 2). The X-ray crystal structure belonging to the *P*2_1_ space group was solved by molecular replacement using the PDB 3MIN as a template. The structure was refined to a resolution of 3.03 Å and contained 2 MoFe proteins in the asymmetric unit (Table S2). The cell dimensions did not fit any of the previously solved structures (Table S3) and might be due to the introduced mutations that impacted the crystal packing (Figure S2, Supporting Information). The α-C45A/L158C MoFe protein was found to overlay well with high-resolution structures previously reported for wild-type MoFe protein from *A. vinelandii*, suggesting a minimal impact of the introduced mutations on the overall conformation of the MoFe protein (Table S4 and Figure 2A).^23,24^ Despite the rather low resolution, some distinct changes in the rotamers of amino acids around the introduced mutations were also observed (Figure 2BC), once again being consistent with the mutations having been successfully introduced. Importantly, the P cluster and FeMoco of the α-C45A/L158C MoFe protein were both found to be present and intact (Supporting Information, Figures S3).

### Desthiobiotin-Maleimide Steric Inhibitor

As depicted in Figure 1, the α-L158C mutation is positioned at the Fe protein-binding interface on the MoFe protein. To interrogate the half-reactivity of nitrogenase’s MoFe protein, we sought to modify only one-half of the α-C45A/L158C MoFe protein at this position with a steric inhibitor that would enable both (i) inhibition of the Fe protein on this half of the MoFe protein and (ii) affinity purification of this hybrid α-C45A/L158C MoFe protein.

**Figure 3:**
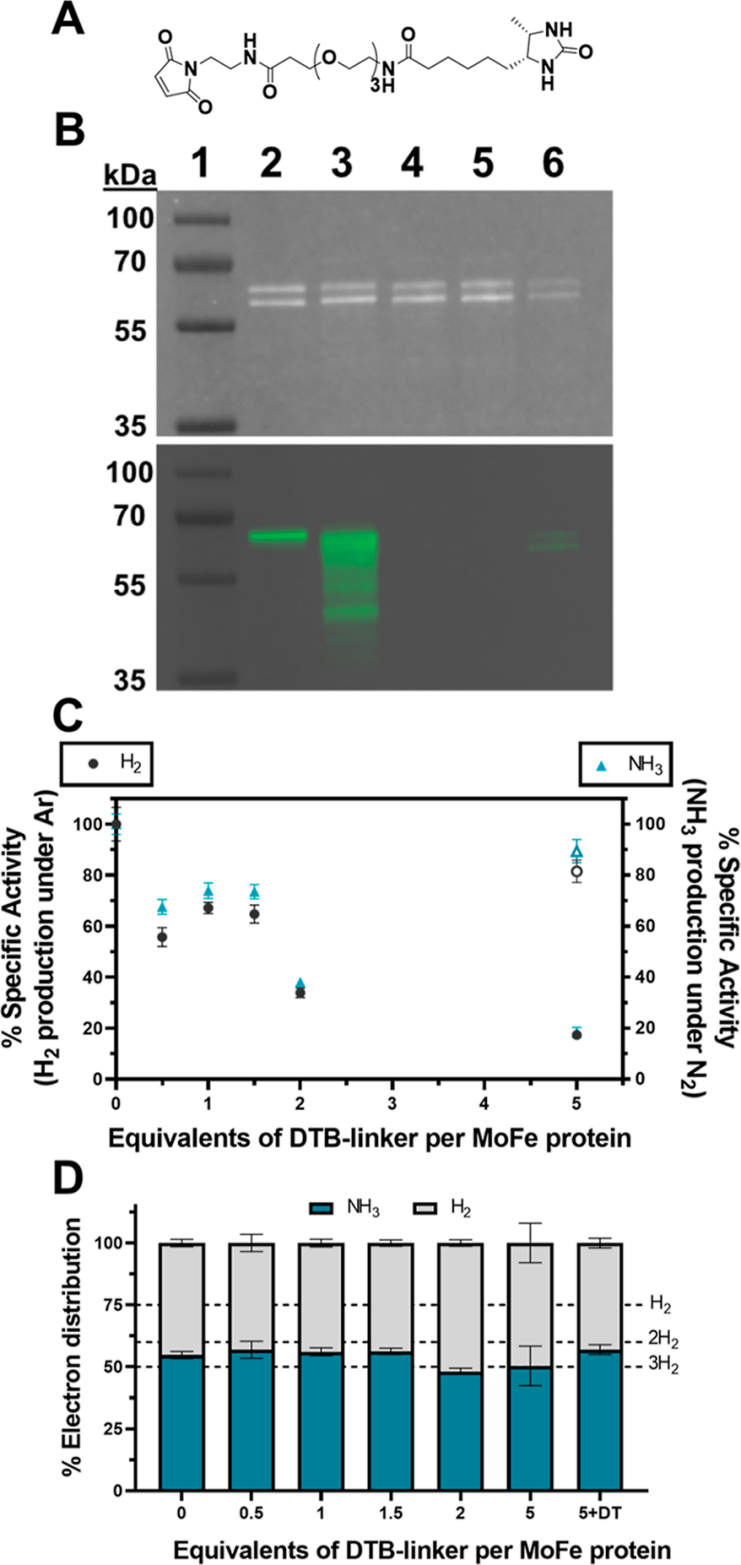
Analysis of maleimide-based DTB steric inhibitor reaction with MoFe protein. (A) Structure of the maleimide-DTB inhibitor. (B) SDS-PAGE analysis (top, stain-free total protein imaging of the PVDF membrane) and western blotting with Streptavidin-HRP (bottom) of α-C45A/L158C MoFe proteins after treatment with the DTB inhibitor. Lanes: 1 = molecular weight marker; 2 = Strep-tagged WT MoFe protein (*N*_term_ Strep-II tag on α-subunit); 3 = α-C45A/L158C MoFe protein treated with DTB inhibitor (DT-free); 4 = “3” although the reaction was performed in the presence of 1 mM DT, 5 = “3” although the reaction was performed in the presence of 10 mM DT, 6 = “3” although the reaction was performed in the presence of 1 mM TCEP. 1 μg protein per well. (C) Residual specific activities with increasing equivalents of DTB inhibitor per one α-C45A/L158C MoFe (left *y* axis labeled as black dots: for H_2_ evolution under 1 atm Ar; right y axis labeled as blue triangles: NH_3_ evolution under 1 atm N_2_). Empty symbols for α-C45A/L158C treated with 10 mM DT before DTB inhibitor addition. All activity assays were performed for 8 min at 30 °C with 0.1 mg mL^–1^ MoFe protein and 16.6 molar equivalents of the Fe protein. (D) Percentage electron distribution between H_2_ formation (gray, 2*e*^–^ per H_2_) and N_2_ fixation (turquoise, 3*e*^–^ per NH_3_) under 1 atm N_2_ with increasing equivalents of the DTB inhibitor. (C,D) *n* = 3 and error bars represent SD (propagated where necessary).

Previously, iodoacetamide-Cys reactivity was employed to attach a Ru-based photosensitizer to this α-C45A/L158C MoFe protein.^19^ In this work, we elected to utilize maleimide-Cys thiol-Michael addition chemistry to modify the α-158C residue due to its improved chemoselectivity over iodoacetamides.^25^ Initially, a steric inhibitor was synthetized by coupling a poly(ethylene glycol)_3_-modified desthiobiotin moiety with *N*-aminoethylmaleimide via a peptide/amide bond formation (Figure 3A; additional details are provided in the Supporting Information, Figures S4–S6). The maleimide moiety was incorporated for the site-selective modification of the α-C158 residue of the α-C45A/L158C MoFe protein, whereas the desthiobiotin moiety was included as a binding motif for avidin-based affinity purification post conjugation of the MoFe protein. It was hypothesized that the poly(ethyleneglycol) repeating units would both increase the size-in-space of the steric inhibitor (and therefore its potency) and increase its solubility during protein conjugation. Initially, this maleimide-modified desthiobiotin-containing inhibitor (referred to subsequently as “DTB”) was incubated with both wild-type and α-C45A/L158C MoFe proteins, and western blotting with a Streptavidin-horseradish peroxidase (HRP) conjugate to confirm successful modification of the MoFe proteins (Figures 3B and S7–S8).

Sodium dithionite (DT) is commonly used as a reducing agent during the purification and handling of nitrogenases due to their deactivation by molecular oxygen (O_2_). We hypothesized that, much like thiol-based reducing agents, DT could reduce the maleimide functional group of DTB and thus quash our Cys functionalization strategy. Therefore, DT-free MoFe protein samples were prepared (Supporting Information) and Cys-maleimide labeling was evaluated in the presence and absence of DT.^26^ As qualitatively shown in Figure 3B, the presence of DT in the MoFe protein samples (additional DT was not included during the reaction) was observed to lower the overall labeling of the MoFe proteins with DTB (also Figure S8, Supporting Information). Importantly, this issue could not be completely resolved with the use of tris(2-carboxylethyl)phosphine (TCEP) as a replacement reducing agent, commonly used in thiol-maleimide Michael addition reactions.

DT was therefore removed from all subsequent MoFe protein preparations prior to maleimide functionalization reactions, only being reintroduced to terminate the maleimide-Cys reaction and maintain reducing conditions after the incubation period. Importantly, the omission of DT from the purification procedure (removed during the first purification column) did not result in a statistically lowered specific activity of the α-C45A/L58C MoFe protein (*t*-test, *P* = 0.93).

We next evaluated the residual activities of the α-C45A/L158C MoFe protein (DT-free) following incubation with varying molar equivalents of the DTB inhibitor, using the Fe protein as the electron donor for H^+^ reduction under Ar and N_2_ reduction (Figure 3C; specific activities are provided in Figure S9). Although a marked decreased in activity was observed globally, the residual activities of the α-C45A/L158C MoFe proteins treated with only 0.5–1.5 molar equivalents of DTB were found to range from approximately 55–75%, indicating that DTB effectively inhibits the Fe protein and subsequent substrate reduction by the MoFe protein. Increasing molar equivalents of DTB were found to further decrease the specific activity of the MoFe protein (<20% with 5 molar equivalents). As previously shown, 10 mM DT significantly impeded the Cys labeling of the α-C45A/L158C MoFe protein with the DTB inhibitor, confirming the necessity to remove DT from the α-C45A/L158C MoFe protein prior to the reaction. DTB steric inhibition was also performed on the WT MoFe protein (exploiting the α-C45 residue), which exhibited a less pronounced decrease in specific activity (Figure S9, Supporting Information).

**Figure 4:**
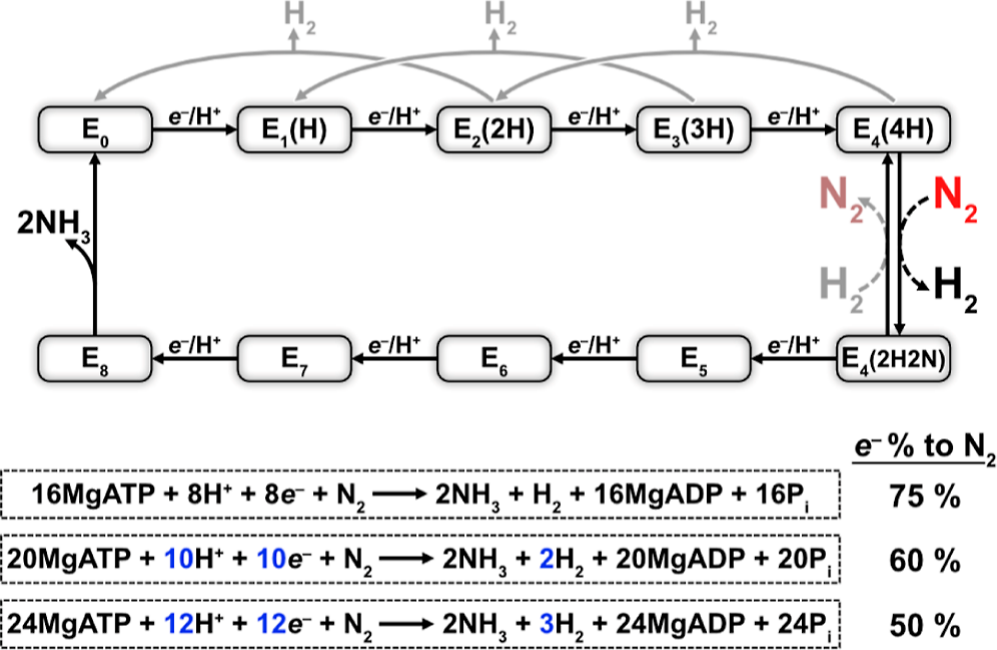
Simplified Lowe-Thorneley scheme for nitrogenase. N_2_ associates to the FeMoco at the E_4_ state along with the reductive elimination of H_2_ by a productive pathway. Non-productive H_2_ release by the FeMoco is colored in gray. The release of 2NH_3_ is placed after the E_8_ state in this representation. Percentage electron distribution is calculated assuming that 2*e*^–^ are required for 2H^+^ reduction to H_2_ and that 6*e*^–^ are required for N_2_ reduction to 2NH_3_.

Having observed a decrease in both H_2_ formation (under Ar and N_2_) and NH_3_ formation (under N_2_), we next determined whether the steric inhibition on one αß half of the α-C45A/L158C MoFe protein impacts the distribution of electrons between H^+^ and N_2_ reduction (i.e., does a cooperativity mechanism contribute to nitrogenase’s selectivity toward N_2_ fixation?). According to the modified Lowe-Thorneley model of nitrogenase’s enzymatic mechanism, the reductive elimination of one H_2_ enables the binding and subsequent reduction of each N_2_ at the FeMoco, leading to the reaction stoichiometry in eq 1 (Figure 4).^1,10^ This has been experimentally observed, with H_2_ production remaining persistent under a high pressure of N_2_ (50 atm).^7^ The 6*e*^–^ reduction of N_2_ therefore requires a total of 8*e*^–^ (the reductive elimination of H_2_ requires 2*e*^–^), with 75% of the electrons delivered to the MoFe protein ultimately being detected as NH_3_. However, in practice under typical laboratory conditions (i.e., 1 atm of N_2_), the distribution of electrons toward N_2_ fixation typically reaches an upper limit of 60%.^20,27−30^ This can be explained by the non-productive release of H_2_ from reduced FeMoco states by the protonolysis of metal-hydrides, which also explains nitrogenase’s H_2_-production activity in the absence of N_2_ (Figure 4).^8^ Here, we observed an electron distribution between 50 and 60% for N_2_, implying that at least 2 or 3 H_2_ molecules are released for each N_2_ molecule reduced (presumably including at least one H_2_ reductive elimination step) (Figure 3D). Interestingly, the observed distribution of electrons toward N_2_ fixation remained around 50–60% upon the titration of increasing equivalents of the DTB inhibitor even though the overall specific activities were observed to decrease (Figure 3D). This result provided an initial indication that both halves of the αß MoFe protein are not strictly required to function in order to fix N_2_ in any given αß domain. However, it is necessary to control the homogeneity of the DTB-bound MoFe population to study the cooperativity of MoFe (i.e., to purify half-inhibited MoFe proteins from uninhibited proteins). Therefore, a purification protocol for the half-inhibited MoFe was established.

Although the functionalization of the α-C45A/L158C MoFe protein with the DTB inhibitor was detected by western blotting with a Streptavidin-HRP conjugate (Figure 3B), our attempts to purify this functionalized protein with commercial StrepTactin-based affinity columns (i.e., “StrepTrap” by Cytiva) were unsuccessful. This was hypothesized to be due to poor affinity of this engineered StrepTactin protein for desthiobiotin over the conventional “StrepTag”, and we therefore reoriented our strategy.

## Strep-Containing, Cysteine-Reactive Peptide Permits the Purification of Partially Reactive MoFe Protein Conjugates

**Figure 5:**
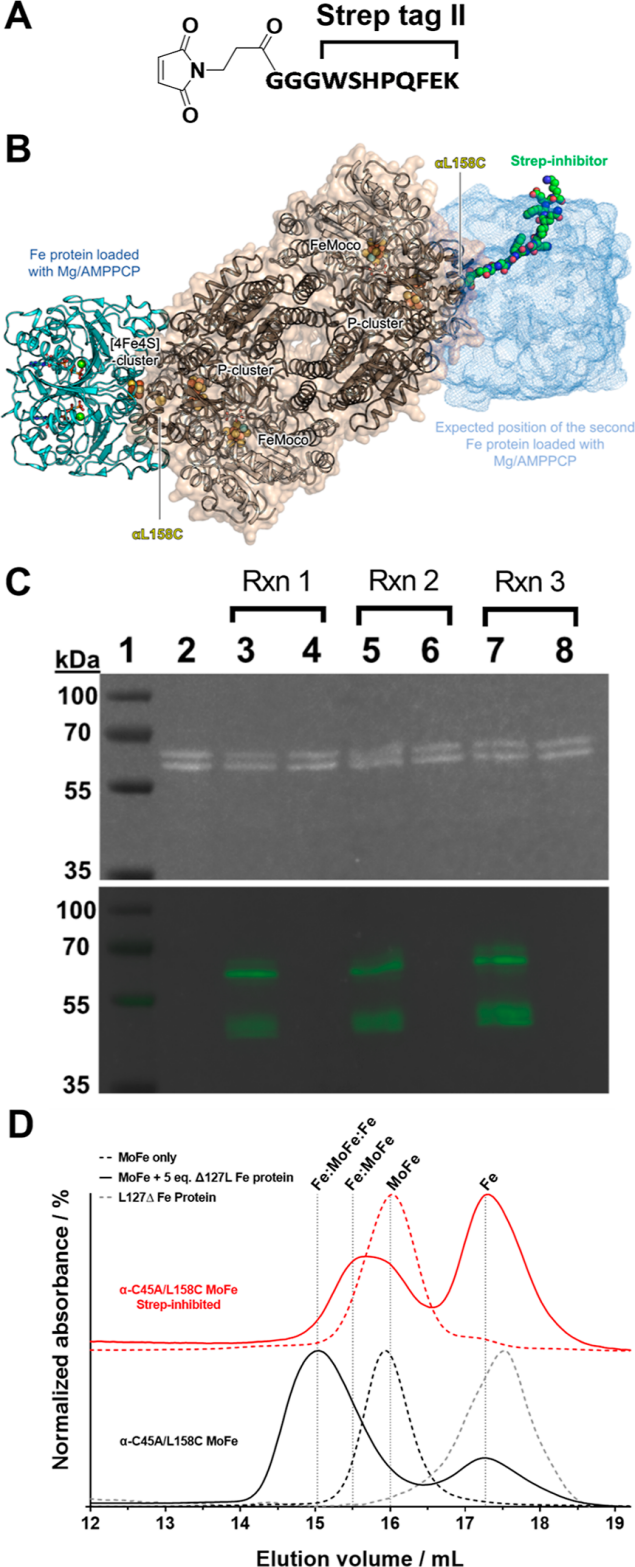
(A) Structure of the maleimide-containing Strep inhibitor. (B) Illustrative model of the Strep inhibitor-modified α-C45A/L158C MoFe protein. (C) SDS-PAGE (upper, stain-free total protein imaging of the PVDF membrane) and western blot (lower) analysis of α-C45A/L158C modification reactions with the Strep inhibitor. Lanes: 1 = molecular weight marker; 2 = untreated α-C45A/L158C MoFe protein; 3/5/7 = Strep-inhibited α-C45A/L158 MoFe protein (eluted from the StrepTactin solid phase); 4/6/8 = unmodified α-C45A/L158C MoFe protein that did not tightly bind to the column solid phase (flow-through). 3/4, 5/6, and 7/8 represent three independent functionalization reactions. Western blotting was performed using a StrepTactin-HRP conjugate (artificially colored image). 0.5 μg protein per well. (D) Gel-filtration of untreated (black dashed line) and Strep-inhibited (red dashed line) α-C45A/L158C MoFe protein. These MoFe proteins were also analyzed after pre-treatment with 5 molar equivalents of L127Δ Fe protein (solid lines) in order to form α-C45A/L158C MoFe:Fe protein complexes.

We next sought to replace the DTB inhibitor with an alternative steric inhibitor that would additionally enable affinity-based purification. Given the widespread success of “Strep-tag” peptides for affinity purification, we elected to employ an N-terminal-modified Strep-tag peptide for the purification of α-C45A/L158C MoFe proteins that had been successfully modified (sequence: maleimide-GGGWSHPQFEK, referred to herein as the “Strep” inhibitor) (Figure 5AB).

The Strep inhibitor was reacted with α-C45A/L158C MoFe with a 0.5:1 molar equivalent of the Strep/α-C45A/L158C MoFe protein (four accessible Cys158 residues per Strep-maleimide) to favor the formation of the half-functionalized MoFe protein over the doubly inhibited MoFe protein (an additional discussion can be found in the Supporting Information, Figure S10). After 4 h of reaction, the maleimide was quenched by the addition of 1 mM DT, and the mixture was loaded onto a commercial pre-packed StrepTactin column (Figure S11, Supporting Information). Unmodified (uninhibited) MoFe protein was collected in the flowthrough fraction, and a dark band was observed to bind to the top of column, consistent with the successful functionalization of the α-C45A/L158C MoFe protein. This functionalization reaction was performed in triplicate on the same sample of the α-C45A/L158C MoFe protein. Figure 5C highlights the purity of the uninhibited (flow-through) and Strep-inhibited MoFe proteins (StrepTactin-bound), where western blotting with a StrepTactin-HRP conjugate highlighted the presence of the Strep moiety on only the inhibited (modified) MoFe protein samples. A lower molecular weight impurity with high affinity to the StrepTactin conjugate was identified, although this was not expected to impact subsequent studies of the Strep-inhibited MoFe proteins due to its comparatively low abundance on the SDS-PAGE gel (this was also not identified during subsequent proteomics analysis discussed below). Quantification of the total Strep-inhibited MoFe protein fraction revealed a functionalization efficiency of 14% ± 1; this reflects the inefficiency of the maleimide-Cys labeling reaction that may be in part due to the inward-facing geometry of the solvent-exposed α-L158C residue, which further reduces the probability of obtaining di-functionalized MoFe proteins (Figure 2C).

LC-ESI-MS/MS was performed on a sample of the Strep-inhibited α-C45A/L158C MoFe protein to confirm the presence of the maleimide-Strep modification on the intended α-C158 residue. LC-ESI-MS/MS analysis of the SDS-PAGE-excised sample (81% α-subunit coverage, 91% ß-subunit coverage) confirmed that the Strep-modification was principally performed on the peptide fragment (trypsin-digested) containing the α-C158 residue, although an additional P cluster-coordinating Cys residue is also present on this fragment (α-C154). A potential functionalization was also identified on the ß-subunit/NifK (a single partially solvent-exposed peptide fragment), although this low-score hit could not be fully assigned (further discussion in the Supporting Information). Thus, the WT MoFe protein incubated with an excess of the Strep-maleimide inhibitor per MoFe (5 molar equivalents, subsequently purified over a StrepTactin column) was also analyzed by LC-ESI-MS/MS (88% α-subunit coverage, 92% ß-subunit coverage). The Strep-inhibitor modification was not detected on the α-C154-containing fragment and is consistent with good selectivity of the Strep inhibitor to the surface-exposed α-C158 residue. Further, the potential modification on the ß-subunit was not identified, further supporting its identification on the α-C45A/L158C MoFe protein to be a false positive due to its low-quality spectra (further discussion in the Supporting Information). While our data are indicative of partial MoFe protein labeling, it is not possible to quantify the fraction of half-inhibited MoFe (the target) vs doubly inhibited MoFe with this approach.

Anoxic native-PAGE analysis was performed to confirm that the Strep-inhibited α-C45A/L158C MoFe protein retained its heterotetrameric organization (Figure S12, Supporting Information). Interestingly, the unmodified MoFe protein appears as a major product at ∼240 kDa although a faint product with a slightly lower molecular weight was also identified systematically during repeated native-PAGE analysis of both the WT and α-C45A/L158C proteins. Due to the high purity of the MoFe proteins (by SDS-PAGE analysis), we hypothesized that this second product was due to a different conformation of the MoFe proteins that was realized predominantly during electrophoretic analysis. Different conformations of MoFe proteins have previously been observed during native-PAGE analysis.^31^ Interestingly, Strep-inhibited MoFe protein samples (WT and α-C45A/L158C) qualitatively exhibited more of this second conformation than the proteins that did not bind to the StrepTactin solid phase during purification. Subsequent limited proteolytic analysis (Figure S13, Supporting Information) indicated that uninhibited and Strep-inhibited α-C45A/L158C MoFe did not exhibit a significant difference in their conformational flexibilities, and we concluded that this additional conformational state is therefore indeed induced during electrophoretic analysis, which is further pronounced following the anchoring of this 1.4 kDa Strep-inhibitor peptide to the α-subunit of the MoFe protein (in either the WT α-C45 or α-C158 position).

These results were confirmed by gel filtration in which uninhibited and Strep-inhibited α-C45A/L158C proteins share very similar elution volumes (Figure 5D). We also used this method to investigate whether a Fe_2_:MoFe transition-state complex could be formed between the Strep-inhibited α-C45A/L158C MoFe protein and the Fe protein as evidence for inhibition of the Fe protein association in proximity to the P cluster site on the MoFe protein (Figure 5D). A non-ATP-hydrolyzing L127Δ Fe protein mutant was employed, which was previously found to form a tight complex with the MoFe protein.^32^ To confirm the possibility of the L127Δ Fe protein to associate to the α-C45A/L158C Strep-inhibited MoFe protein, both proteins were incubated and the complex was subsequently purified using the N-terminal His-tag on the α-subunit of the MoFe protein. SDS-PAGE analysis of the obtained sample confirmed the presence of protein bands corresponding to both the MoFe and Fe proteins (Figure S14, Supporting Information). After incubation of the uninhibited α-C45A/L158C MoFe protein with 5 molar equivalents of the L127Δ Fe protein, virtually all of the MoFe protein was found to shift to a complex of increased molecular weight, consistent with the formation of a tight Fe_2_:MoFe complex (Figure 5D). On the other hand, incubation of the Strep-inhibited α-C45A/L158C MoFe protein with the L127Δ Fe protein resulted in a broadened MoFe protein peak; further, a comparatively increased quantity of the non-complexed L127Δ Fe protein was observed. We hypothesize that this broadening corresponds to a mixed population of (i) non-complexed Strep-inhibited MoFe protein, (ii) Fe_1_:MoFe protein complex, and (iii) doubly inhibited MoFe protein, consistent with inhibition of Fe protein association to the MoFe protein in the presence of the Strep-inhibitor at the α-C158 position. Multi-Gaussian peak analysis of this gel-filtration profile was performed in an attempt to provide indicative quantification of these different fractions (Figure S15). We calculated that this broad feature may comprise ∼68% of the half-inhibited MoFe:Fe_1_ complex and ∼32% of either non-complexed half-inhibited MoFe or doubly inhibited MoFe (we cannot discriminate between the latter two). Recent cryogenic electron microscopic investigation into Fe:MoFe complex formation during turnover suggests that the MoFe protein preferentially docks with only one Fe protein at a time.^17^

The formation of a Fe_2_:MoFe protein complex (uninhibited and Strep-inhibited) was also evaluated by native-PAGE analysis, also suggesting that the Strep-inhibited α-C45A/L158C MoFe protein does not form a tight Fe_2_:MoFe complex (Figure S16, Supporting Information). Uninhibited WT and α-C45A/L158C MoFe proteins were observed to form two different complexes of an apparent larger size. Keeping in mind the proposed alternative conformation of the Strep-inhibited α-C45A/L158C MoFe protein during native-PAGE analysis, incubation with 5 molar equivalents of the L127Δ Fe protein yielded one major (and one significantly weaker) complex of apparent increased size, consistent with inhibition of the Fe protein access to primarily one-half of the MoFe protein.

### Reactivity of the Strep-Inhibited MoFe Protein

After having successfully purified the Strep-inhibited α-C45A/L158C MoFe protein, we next evaluated its remaining residual specific activity. Importantly, the mean specific activities for both H_2_ (1 atm Ar) and NH_3_ (1 atm N_2_) production of the MoFe proteins that did not react with the Strep-inhibitor (the flow-through fractions) were found not to differ from the unreacted control protein (one-way ANOVA, *P* = 0.0833 and 0.3323), indicating that neither the reaction conditions nor the handling of the samples drastically impacted the enzymatic activity.

**Figure 6:**
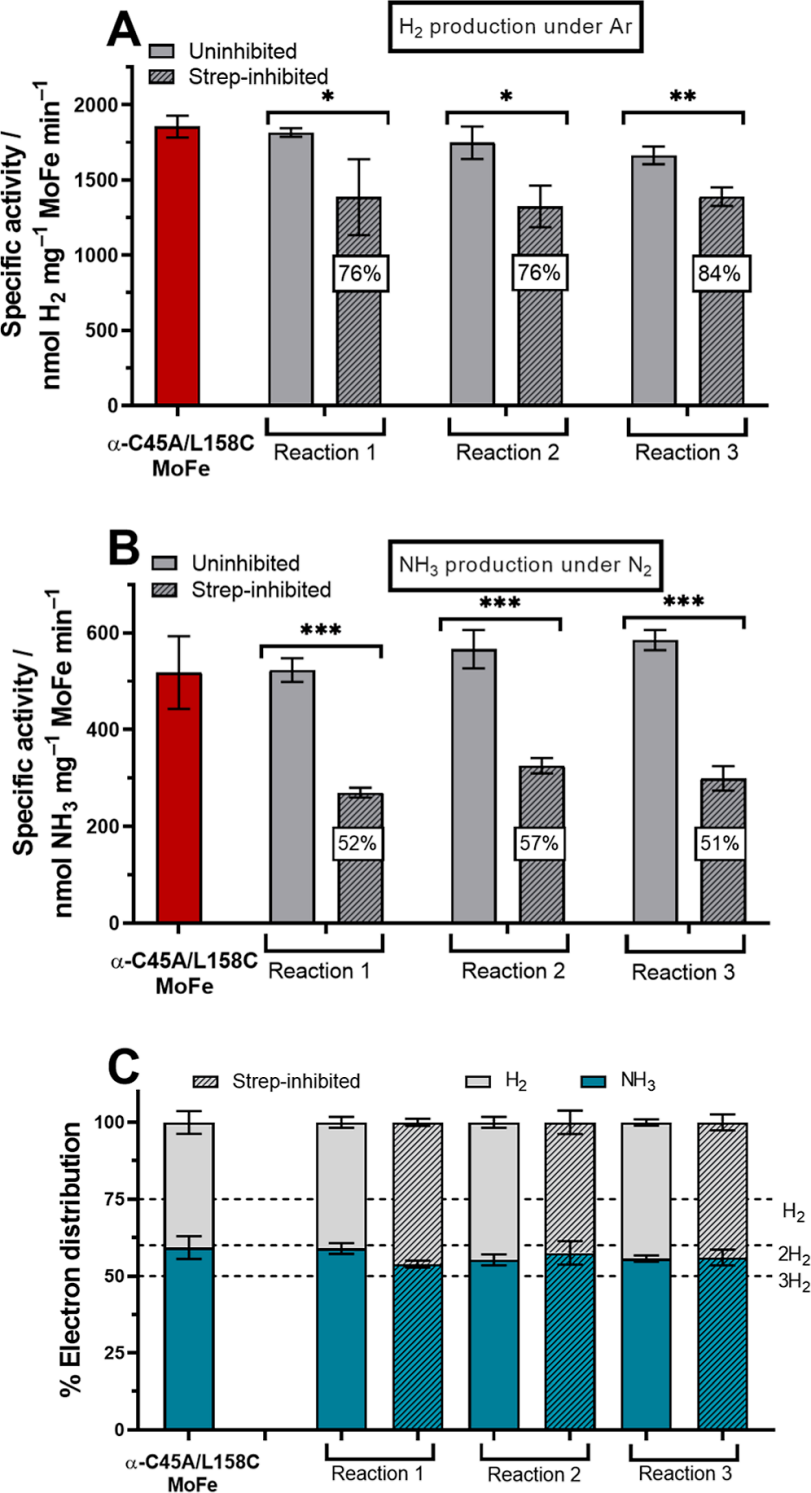
(A) Specific activity for H_2_ evolution under 100% Ar headspace. Red is the standard α-C45A/L158C MoFe purified in the absence of DT. Light gray bars represent the activities obtained for the α-C45A/L158C MoFe protein from the Strep-inhibitor functionalization reactions that did not bind to the StrepTrap XT solid phase. The hatched bars represent Strep-inhibited α-C45A/L158C MoFe (eluted from the StrepTrap XT solid phase). The percentage value in the white square corresponds to the specific activity of Strep-inhibited MoFe divided by specific activity to the related unreacted MoFe or FT fraction. (B) Specific activity for NH_3_ evolution under a 100% N_2_ atmosphere. (C) Electron distribution in percentage. Blue is for electrons consumed to make NH_3_ (3*e*^–^ per NH_3_) and gray is for electrons consumed to make H_2_ (2*e*^–^ per H_2_) under a N_2_ atmosphere. All activity assays were performed for 8 min at 30 °C with 0.1 mg mL^–1^ MoFe protein and 16.6 molar equivalents of the Fe protein. (A–C) *n* = 3 and error bars represent SD (propagated where necessary). Significance: ns (not significant) for *P* > 0.05, * for *P* ≤ 0.05, ** for *P* ≤ 0.01, and *** for *P* ≤ 0.001.

We then compared the specific activities of the uninhibited and Strep-inhibited α-C45A/L158C MoFe proteins for H^+^ reduction under 1 atm Ar. As shown in Figure 6A, the specific activities of the Strep-inhibited MoFe proteins were found to be 79 ± 4% (mean of the three reactions) of the specific activities of their uninhibited counterparts. In addition, the specific activities of the three Strep-inhibited α-C45A/L158C MoFe protein samples were not found to significantly differ from one another (H_2_ production under 1 atm Ar, one-way ANOVA, *P* = 0.8755). Our observation of >50% residual H^+^ reduction activity per MoFe protein following Strep-inhibition (where at least one half of each MoFe protein has been inhibited by the Strep-inhibitor) suggests that the uninhibited α-C45A/L158C MoFe protein (more globally, nitrogenase) employs a negative cooperativity mechanism for H^+^ reduction in the steady-state/continued turnover. This is consistent with the previous observation that the electron delivery cycle of the Fe protein also experiences negative cooperativity (cooperativity in terms of product formation in the steady state was not evaluated).^12^

Another recent study observed that the MoFe protein does not exhibit cooperativity when producing H_2_ under Ar.^13,33^ As mentioned in the introduction, this approach inhibited Fe protein access to one αß half by introducing the L127Δ Fe protein to competitively form a non-dissociating complex (or by using a mismatched Fe protein from a different organism). Our gel filtration data (Figure 5D) indicate that MoFe proteins half-inhibited by the L127Δ Fe protein may, in fact, not form non-dissociating complexes on only one αß half of the MoFe protein, leading to a population of Fe_2_:MoFe and free MoFe proteins (i.e., in the case of a 1:1 Fe_L127Δ_:MoFe protein ratio). The Strep-inhibition approach reported here is not anticipated to introduce a long-range conformational change at the second Fe protein-binding site. Further, we observed that the affinity of the Fe protein to the MoFe protein is relatively unchanged (H_2_ production under 1 atm Ar) in the presence of the Strep inhibitor on the MoFe protein (Supporting Information, Figure S17).

The specific activity for NH_3_ formation under 1 atm N_2_ was investigated after Strep-inhibition of the MoFe protein, wherein a residual specific activity of 53 ± 4% (mean of the three reactions) was observed in comparison to that of the uninhibited MoFe protein (Figure 6B, and Figure S18, Supporting Information). The specific activities of these three functionalization reactions were found to differ for NH_3_ production only weakly under 1 atm N_2_ (one-way ANOVA, *P* = 0.0467). As shown in Figure 6C, the % electron distribution toward N_2_ fixation remains between 50 and 60% for all three Strep-inhibited MoFe proteins under 1 atm N_2_ (one-way ANOVA, *P* = 0.1896), confirming that under these conditions nitrogenase’s selectivity toward N_2_ on both αß halves is not a result of cooperative behavior across the MoFe protein. The determination of whether a cooperativity mechanism is at play during N_2_ fixation (in terms of specific activity) is more delicate and is treated in the following section.

**Figure 7:**
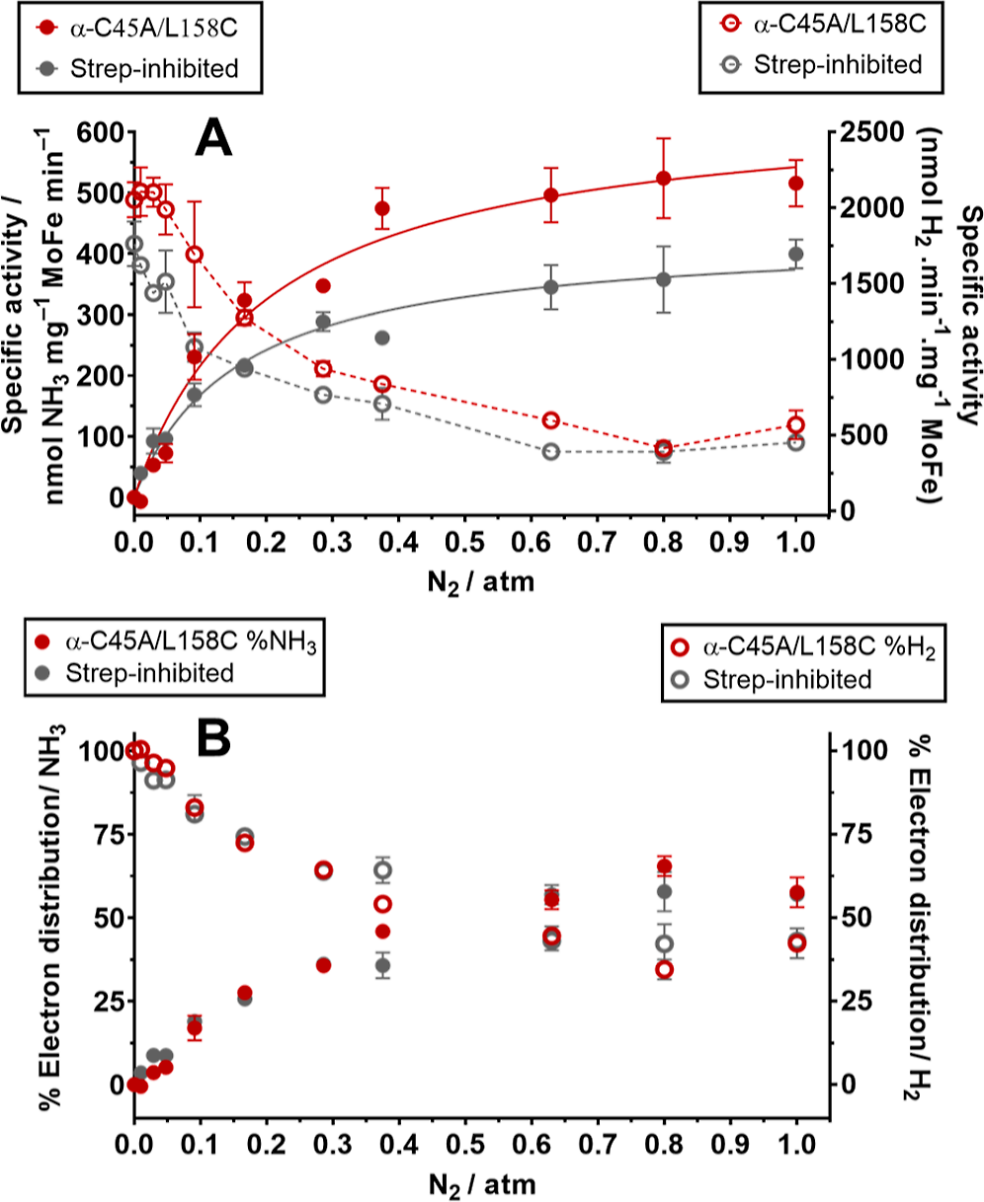
(A) Specific activity for H_2_ and NH_3_*versus* N_2_ partial pressure. The left axis represents specific activity in nmol NH_3_ min^–1^ mg^–1^ MoFe (solid lines, solid points). The right axis represents specific activity in nmol H_2_ min^–1^ mg^–1^ MoFe (dashed lines, hollow points). Red data: uninhibited α-C45A/L158C MoFe; gray data: Strep-inhibited α-C45A/L158C MoFe. (B) Percentage of electron distribution toward N_2_ fixation (solid points) or H_2_ production (hollow points). Red data: uninhibited α-C45A/L158C MoFe; gray data: Strep-inhibited α-C45A/L158C MoFe. Percentage electron distributions are determined by the assumption that H_2_ formation requires 2*e*^–^ and N_2_ fixation requires 6*e*^–^. All activity assays were performed for 8 min at 30 °C with 0.1 mg mL^–1^ MoFe protein and 16.6 molar equivalents of the Fe protein. (A,B) *n* = 3 (Technical repeats) and error bars represent SD (propagated where necessary); *n* = 2 (technical repeats) for 0.8 atm N_2_ data points.

A Strep-inhibition reaction was performed on a larger batch of α-C45A/L158C MoFe protein (∼18 mg of Strep-inhibited protein obtained) to evaluate Michaelis–Menten kinetic parameters and the distribution of electrons toward N_2_ fixation under a range of N_2_ partial pressures (Figure 7A). Neither the Michaelis constant (*K*_M_) nor maximum velocity (*V*_max_) for the α-C45A/L158C MoFe protein was found to differ statistically from the wild-type MoFe protein (*P* = 0.2065 and *P* = 0.3873, Figure S19/Table S5, Supporting Information). Importantly, the *V*_max_ for the Strep-inhibited α-C45A/L158C MoFe protein was found to be 66% of that of the uninhibited MoFe protein, while the affinity toward N_2_ was unchanged (*P* = 0.2457, Table 1). The *k*_cat_ normalized per FeMoco was observed to significantly increase following Strep-inhibition of the MoFe protein from 1.3 to 1.7 s^–1^, *P* = 0.0102; the possible presence of a doubly inhibited MoFe protein in the sample suggests that the determined value of 1.7 s^–1^ per FeMoco could indeed be larger.

**Table 1:** Michaelis–Menten Kinetic Parameters for α-C45A/L158C and Strep-Inhibited α-C45A/L158C MoFe Proteins

Michaelis–Menten parameter (N_2_ fixation)	α-C45A/L158C	α-C45A/L158C Strep-inhibited
*K*_M_^app^ (atm)	0.20 ± 0.05	0.16 ± 0.03
*V*_max_^app^ (nmol NH_3_ min^–1^mg^–1^)	651 ± 55	432 ± 29
*k*_cat_ (s^–1^)	1.3 ± 0.1	>1.7 ± 0.1^a^
*k*_cat_/*K*_M_ (s^–1^ atm^–1^)	6.5 ± 1.7	11.0 ± 2.5
*k*_cat_/*K*_M_ (x10^4^ s^–1^ M^–1^)	1.0 ± 0.3	1.7 ± 0.4

a *k*_cat_ is defined here as turnover frequency per active αß half (nmol NH_3_ FeMoco^–1^ s^–1^). The possible presence of a doubly inhibited MoFe protein in this sample could result in a larger *k*_cat_ value per FeMoco (αß half). Partial pressures of N_2_ were from atm to M^–1^ using a Henry’s law conversion factor of 6.4 × 10^–4^.^34^

Importantly, the quantity of H_2_ produced under increasing pressures of N_2_ is consistent with increased N_2_ fixation by nitrogenase (electron allocation toward N_2_ fixation), with uninhibited and Strep-inhibited MoFe proteins exhibiting similar trends. Figure 7B reports the percentage *e*^–^ distribution between N_2_ fixation and H_2_ formation by uninhibited and Strep-inhibited α-C45A/L158C MoFe proteins with increasing N_2_ pressures. A maximum electron distribution of approximately 65% toward N_2_ fixation was observed under these conditions, with no clear difference in electron distribution between uninhibited or inhibited α-C45A/L158C MoFe observed over this range of N_2_ pressures. In contrast to the *k*_cat_ and *k*_cat_/*K*_M_ parameters, this suggests that a cooperativity mechanism, or the arrangement of the MoFe protein as an α_2_ß_2_ heterotetramer, does not contribute toward the selectivity of nitrogenase toward N_2_ under these conditions.

**Figure 8:**
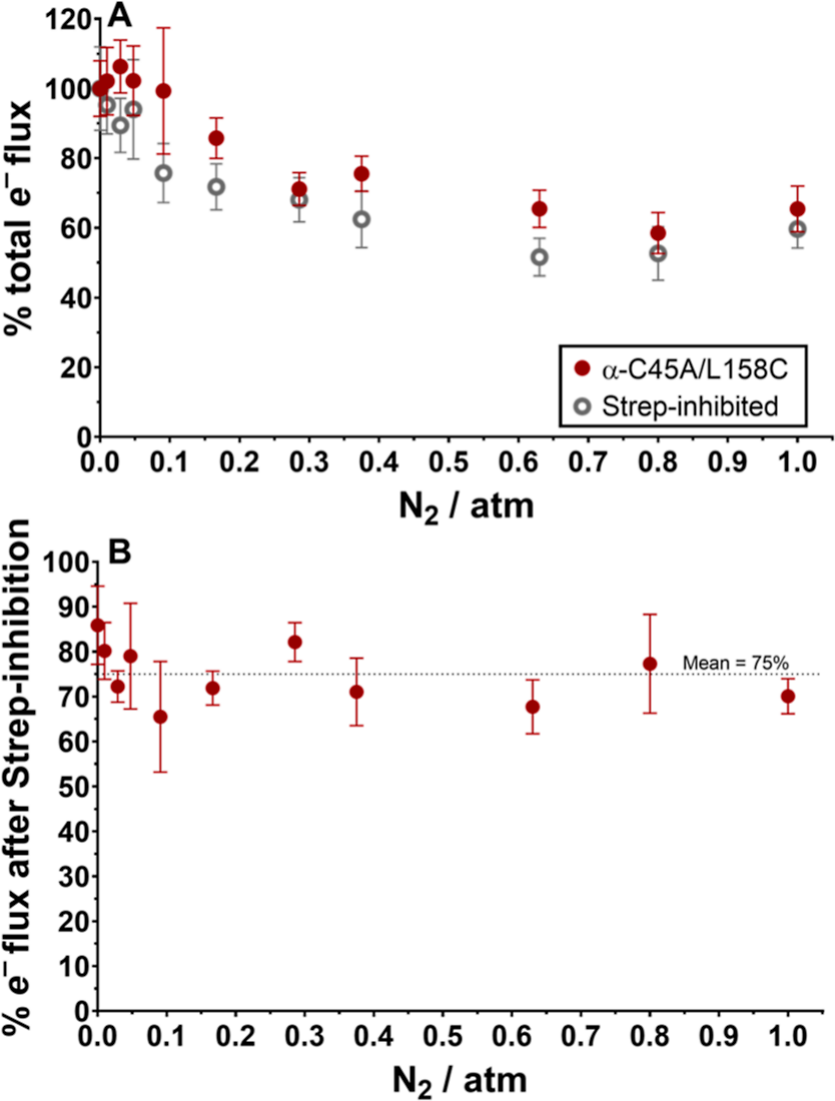
(A) Percentage total electron flux of uninhibited and Strep-inhibited α-C45A/L158C MoFe proteins upon the introduction of increasing N_2_ partial pressures, with 1 atm Ar resulting in the largest total electron flux for both proteins. (B) Remaining total electron flux after Strep-inhibition of the α-C45A/L158C MoFe protein between 0 and 1 atm of N_2_. Percentage electron distributions are determined by the assumption that H_2_ formation requires 2*e*^–^ and N_2_ fixation requires 6*e*^–^. All activity assays were performed for 8 min at 30 °C with 0.1 mg mL^–1^ MoFe protein and 16.6 molar equivalents of the Fe protein. (A,B) *n* = 3 and error bars represent SD (propagated where necessary).

In agreement with previous reports, the total electron flux (total electrons consumed determined by production quantification, where H_2_ = 2*e*^–^ and NH_3_ = 3*e*^–^) of uninhibited and Strep-inhibited α-C45A/L158C MoFe proteins was observed to decrease following the introduction of increasing N_2_ partial pressure in the reaction vials (Figure 8A, 100% electron flux represents activity under 1 atm Ar).^27−29^ However, no clear difference in the decrease of electron flux with or without Strep-inhibition over 0–1 atm of N_2_ was observed (electron flux decreases uniformly for uninhibited and strep-inhibited α-C45A/L158C MoFe protein), where Figure 8B compares the percentage remaining total electron flux over 0–1 atm N_2_ after having Strep-inhibited the α-C45A/L158C MoFe protein (in comparison to the uninhibited α-C45A/L158C MoFe protein). Importantly, total electron flux remains <100% regardless of the N_2_ partial pressure (mean = 75%), suggesting that the MoFe protein indeed follows a negative cooperativity mechanism with respect to electron delivery. This is consistent with the earlier finding that the Fe protein electron delivery cycle exhibits negative cooperativity in the pre-steady state.^12^ We therefore conclude that negative cooperativity for electron delivery does in fact propagate itself in the formation of total products (including the production of NH_3_), although this does not significantly impact the selectivity of N_2_ fixation by this enzyme complex. Considering the proposal that negative cooperativity may arise from the MoFe protein only interacting with one Fe protein at a time during turnover alongside our observations of >50% activity for Strep-inhibited MoFe proteins, we hypothesize that negative cooperativity is introduced after the association of a second Fe protein to a Fe_1_:MoFe protein turnover complex.^17^

Badalyan et al. employed voltammetry to determine the rate constant for electron consumption by nitrogenase to be 14 s^–1^; importantly, this value was observed to remain constant in the presence of N_2_, in fact suggesting that electron flux to nitrogenase remains constant.^30^ In this study, 23% of electrons were unaccounted for (not detected as H_2_ or NH_3_). More recently, Lee et al. observed that nitrogenase proteins purified in the strict absence of DT were unable to undergo continued turnover, where it was proposed that sulfite (resulting from DT decomposition/oxidation) plays an additional sulfur-recharging role upon reduction at the FeMoco.^26^ Such a reaction may explain the apparent decrease in total electron flux upon N_2_ fixation by nitrogenase.

## Conclusions

Long-range (>95 Å) communication between the Fe proteins interacting with the two αß halves of nitrogenase’s MoFe protein is thought to be of mechanistic importance.^14^ Previous studies have observed (i) negative cooperativity for electron delivery in the pre-steady-state, and (ii) no cooperativity for H^+^ reduction by nitrogenase upon “locking” one αß MoFe half in an inactive Fe protein-bound state.^12,13^

We sought to inhibit one αß half of the MoFe protein by an approach that was not expected to impact the Fe protein transient association behavior on the remaining uninhibited active site. More importantly, we sought to determine whether cooperativity is strictly necessary for N_2_ fixation by nitrogenase. We conclude that negative cooperativity plays a role in both H^+^ reduction to H_2_ and overall electron delivery to the MoFe protein (and, thus, also for N_2_ fixation) and that cooperativity across the MoFe protein is not strictly necessary for the fixation of N_2_ to NH_3_. The *k*_cat_ per FeMoco site for NH_3_ production was observed to increase by 31% upon Strep-inhibition (from 1.3 to >1.7 s^–1^), consistent with negative cooperativity in N_2_ fixation by nitrogenase in terms of electron delivery (selectivity toward N_2_ is not impacted). It remains difficult to precisely determine the magnitude of negative cooperativity-induced suppression of nitrogenase activity in a single αß MoFe protein half due to the possible presence of a doubly inhibited MoFe protein in our Strep-inhibited sample. A potential method to surmount this issue could be the co-expression of two copies of *nifD* (i.e., *nifD* and nifD*) followed by tandem affinity purification.

While an *in vitro* investigation into nitrogenase’s cooperativity mechanism provides insight into its catalytic mechanism, it remains important to determine the importance and magnitude of negative cooperativity of nitrogenase in the context of *in vivo* turnover (in *A. vinelandii*), where (i) Fe/MoFe protein ratios could be more dynamic or (ii) additional partners could play a role (such as maturases, activity-modulating proteins such as CowN,^35^ O_2_-protection proteins such as the Shethna FeSII protein^36,37^ or indeed an as-of-yet unidentified allosteric effector).

## Materials and Methods

### Culturing of *A. vinelandii*

Extensive procedures are reported in the Supporting Information. Briefly, MoFe and Fe nitrogenase proteins were isolated from various *A. vinelandii* strains and were cultivated on a modified Burke’s medium. The *nif* operon was derepressed after overnight growth by centrifugation and resuspension into fresh Burke’s medium lacking fixed N (NH_4_^+^). Cells were harvested by centrifugation after ∼3.5 h and stored at −80 °C until use.

### Fe Protein Purification

All Fe protein purifications were performed in a COY anaerobic chamber (<5% H_2_/>95% N_2_, Michigan, USA). The wild-type Fe protein was purified from a strain derived from *A. vinelandii* DJ that was modified to introduce an 8x His-tag to the N-terminus of NifD (*A. vinelandii* RS1).^20^ The L127Δ Fe protein was purified from the *A. vinelandii* strain DJ1065 (provided by Dennis Dean, Virginia Tech). Cells from a 12 L culture were thawed and resuspended in anoxic lysis buffer (50 mM Tris/HCl, pH 8.0) containing 5 mM DT and 37% v/v glycerol. All Fe protein purification buffers contained 2 mM DT, except for the lysis buffer mentioned above. After incubation for 20 min, the cells were collected by centrifugation and resuspended in glycerol-free lysis buffer to induce cell lysis by osmotic shock. The lysate was incubated on ice for a further 15 min and clarified by centrifugation at 30,000 x *g* (4 °C, 1 h). A post-lysis buffer (2 M NaCl, 234 mM Tris/HCl, 2 mM DT, pH 8.0) was added to the cell-free supernatant to achieve a final NaCl concentration of 0.3 M. The cell-free supernatant was next passed over a HisTrap HP column (5 mL column volume, Cytiva) to remove the His-tagged MoFe protein (below). The column flow-through (containing the Fe protein) was next diluted with a NaCl-free buffer (50 mM Tris/HCl, pH 8.0, 2 mM DT) to obtain a final NaCl concentration of 0.1 M. The Fe protein was first purified over a HiPrep Q-Sepharose HP 16/10 column (20 mL column volume, Cytiva) and eluted over a linear NaCl gradient of 0.2–0.65 M NaCl. Following concentration to <10 mL using a Merck-Millipore stirred concentration cell (30 kDa molecular weight cut-off membrane), the Fe protein was next purified by size-exclusion chromatography over a HiPrep 26/60 Sephacryl S-200 HR column (320 mL column volume, Cytiva) using a Tris running buffer (50 mM Tris/HCl, pH 8.0, 0.5 M NaCl). The eluted Fe protein was concentrated further to >20 mg/mL and flash-frozen in liquid nitrogen until use.

### MoFe Protein Purification

All MoFe protein purifications were performed in a COY anaerobic chamber (<5% H_2_/>95% N_2_, Michigan, USA). The wild-type MoFe protein carrying an 8x His-tag on the N-terminus of NifD was purified from the *A. vinelandii* strain RS1. The α-C45A/L158C MoFe protein was purified from a derivative of *A. vinelandii* RS1 (*A. vinelandii* strain “M1”, Supporting Information), which carried the same 8x His-tag on the N-terminus of NifD. The cell-free supernatant (prepared as above) was first passed over a HisTrap HP column (5 mL column volume, Cytiva). The running buffers for this step (50 mM Tris/HCl, pH 8.0, ±0.3 M imidazole) were incubated overnight in the anaerobic chamber and did not contain DT (all subsequent buffers did not contain DT). MoFe proteins bound to the His-resin were first washed with >3 column volumes of DT-free buffer (0 mM imidazole) to remove DT introduced during cell lysis. After a 20 mM imidazole washing step, MoFe proteins were eluted using 0.3 M imidazole. The eluted MoFe proteins were next diluted with Tris buffer (50 mM Tris/HCl, pH 8.0) to reach a final NaCl concentration of 0.1 M prior to being loaded onto a pre-equilibrated Q-Sepharose FF 16/10 column (20 mL column volume, flow-rate = 20 mL/min). DT-free MoFe proteins were eluted over a linear gradient of 0.2–0.65 M NaCl and concentrated to >20 mg/mL using a 100 kDa stirred concentrator cell (fed with ultra-high-purity N_2_ 5.0) prior to being flash-frozen in liquid nitrogen until use.

### MoFe Protein Functionalization with the DTB Inhibitor

The DT-free MoFe protein was treated with the DTB inhibitor (Supporting Information) freshly prepared in 0.1 M MOPS/NaOH buffer (pH 7.0) for 4 h at room temperature within the COY anoxic chamber. After 4 h, unreacted maleimide was quenched and MoFe proteins were reduced by the addition of DT to a final concentration of 2 mM.

### MoFe Protein Functionalization with the Strep-Tag Inhibitor and Conjugate Purification

A lyophilized synthetic Strep-tag peptide containing an N-terminal maleimide functionality (sequence = GGGWSHPQFEK) was obtained from GenScript (USA) and used as received. This 1.4 kDa peptide functioned as both (i) a steric inhibitor of Fe protein association to the MoFe protein and (ii) a Strep-tag for purification of the MoFe protein conjugates. Strep-inhibited α-C45A/L158C MoFe protein was obtained following the incubation of DT-free α-C45A/L158C MoFe protein with 0.5 molar equivalents of the Strep-tag inhibitor (0.5 inhibitor per MoFe protein, resulting in a 1:4 Strep-tag to surface Cys ratio, freshly dissolved in 0.1 M MOPS/NaOH pH 7.0 to a final concentration of 1–3 mg/mL) for 4 h within the anoxic glovebox. The reaction was quenched (and the MoFe protein reduced) by the addition of DT to a final concentration of 2 mM. The functionalized/inhibited MoFe protein was next purified over a StrepTrap XT column (5 mL column volume, Cytiva) pre-equilibrated with MOPS buffer (0.1 M MOPS/NaOH, 0.2 M NaCl, 2 mM DT, pH 7.0). Unreacted MoFe proteins were collected from the column flow-through, and Strep-inhibited MoFe proteins were collected following the application of 50 mM biotin to the column. MoFe proteins were concentrated as above and flash-frozen in liquid nitrogen until use.

### Nitrogenase Activity Assays

Briefly, nitrogenase activity assays were performed in 13 mL septum-sealed glass vials containing 1 mL of an ATP-regenerating MOPS buffer (100 mM MOPS/NaOH, pH 7.0, 5 mM ATP, 30 mM phosphocreatine, 1.3 mg of bovine serum albumin, 0.2 mg of creatine phosphokinase from rabbit muscle, and 10 mM DT). All activity assays employed DT as the electron donor. All reactions were assembled within an Ar-filled anoxic glovebox (Jacomex, France). MoFe proteins (0.1 mg) and Fe proteins (0.48 mg) were included with a 16.6:1 Fe to MoFe protein ratio, sealed, and vented to atmospheric pressure. The reaction vials were then heated to 30 °C in a shaking water bath and the reaction initiated by the injection of MgCl_2_ to a final concentration of 10 mM using a gas-tight syringe. After 8 min, the reactions were terminated by the addition of 300 μL of 400 mM EDTA. H_2_ was quantified using a gas chromatograph-thermal conductivity detector (molecular sieve 5 Å column, Ar carrier gas, SRI Instruments model 8610C). NH_3_ was quantified by the *ortho*-phthalaldehyde method (corrected to controls and assays performed under 1 atm Ar) using NH_4_Cl as the standard.^20,38^

### Crystallization of the α-C45A/L158C MoFe Protein

The purified enzyme in Tris/HCl buffer (50 mM Tris/HCl, 300 mM NaCl, 2 mM DT, pH 8.0) was crystallized anaerobically at 8 mg/mL^–1^ (under a 100% N_2_ atmosphere) with an OryxNano (Douglas Instrument, UK). The initial screening was performed at 20 °C using the sitting drop method on 96-Well MRC 2-drop crystallization plates in polystyrene (SWISSCI) containing 90 μL of crystallization solution in the reservoir. The protein sample (0.5 μL) was mixed with 0.5 μL of reservoir solution. Crystals were transferred and stored anaerobically in a Coy tent (N_2_/H_2_, 97:3). Thin brown plate crystals appeared after a few weeks. The reservoir solution contained 25% w/v polyethylene glycol 3,350, 100 mM BIS-TRIS at pH 5.5, and 200 mM Lithium sulfate.

### X-ray Crystallography Data Collection and Refinement

Crystal handling was done inside a Coy tent under an anaerobic atmosphere (N_2_/H_2_, 97:3). Crystals were soaked in the crystallization solution supplemented with 15% v/v glycerol as a cryo-protectant before being frozen in liquid nitrogen. Crystals were tested and collected at 100 K at the Swiss Light Source, X06DA–PXIII. Due to the high anisotropy, data were processed and scaled with *autoPROC*.^39^ The relative resolution limits along the unit cell axis are *a* = 4.34 Å, *b* = 4.07 Å, and *c* = 3.03 Å. The molecular replacement was done with *PHASER* from the *PHENIX* package.^40^ The model was then manually built with *COOT* and further refined with *PHENIX* without hydrogens.^41^ The last cycles of refinement were performed with Buster and the final one with *PHENIX*. The model was validated by the MolProbity server (used on the 15th of August 2022).^42,43^

## Supporting Information Available

Protein production and purification methodology; activity assays; analytical gel filtration methodology; limited proteolysis methodology; additional X-ray crystallography data; synthesis of DTB inhibitor; *sacB*-based markerless mutagenesis; additional SDS-PAGE and western blotting; activity assays for the DTB-inhibited wild-type MoFe protein; binding and elution of Strep-inhibited MoFe on StrepTrap XT (Streptactin) columns; native-PAGE; Fe protein titration of the MoFe protein; specific activities for NH_3_ and H_2_ formation by the Strep-inhibited MoFe protein; Michaelis–Menten kinetic parameters for the wild-type MoFe protein. Raw research data and sample reports have been deposited on Zenodo (https://doi.org/10.5281/zenodo.6865680) and contain specific activities, proteomics reports, raw proteomics data, unprocessed X-ray crystallography, sequencing data for *A. vinelandii* strain producing α-C45A/L158C MoFe protein, and sequences of plasmids used to generate this mutant strain (pK18mobsacB) (PDF)

## Notes

One-way ANOVA tests (Brown-Forsythe) and *t* tests were performed using GraphPad Prism. The following notation was employed for statistical significance: ns (not significant) for *P* > 0.05, * for *P* ≤ 0.05, ** for *P* ≤ 0.01, and *** for *P* ≤ 0.001. Graphs were plotted using GraphPad Prism. Figures were created using a combination of Affinity Designer, ChemDraw, ChimeraX, and PyMol. RDM is an Early Career Advisory Board member of JACS Au for 2022. The structural model of the α-C45A/L158C MoFe protein has been deposited on the Protein Data Bank (www.pdb.org), available under the accession code PDB 8BTS.

## Abbreviations

DTB: desthiobiotin-maleimide inhibitor
SDS-PAGE: sodium dodecyl sulfate poly(acrylamide) gel electrophoresis
ATP: adenosine triphosphate
ADP: adenosine diphosphate
FeMoco: FeMo cofactor
DTB: desthiobiotin
DT: dithionite
LC-ESI-MS/MS: liquid chromatographyelectrospray ionization–tandem mass spectrometry.
