# Supplementary material for: Nitrogen Fixation and Hydrogen Evolution by Sterically Encumbered Mo-Nitrogenase
Source: JACS Au. 2023 May 9;3(5):1521–33. doi: 10.1021/jacsau.3c00165 (PMC10207099; doi:10.1021/jacsau.3c00165)
Supplement: Supplementary file 1 — au3c00165_si_001.pdf [file au3c00165_si_001.pdf]

Supporting Information for:

Nitrogen fixation and hydrogen evolution by sterically encumbered Mo-nitrogenase

Cécile Cadoux,[ab] Daniel Ratcliff,[ab] Nevena Maslać[c], Wenyu Gu,[d] Ioannis Tsakoumagkos,[e] Sascha Hoogendoorn,[e] Tristan Wagner[c] and Ross D. Milton[ab*]

[a] Department of Inorganic and Analytical Chemistry, Faculty of Sciences, University of Geneva, Quai Ernest-Ansermet 30, 1211 Geneva 4, Switzerland. [b] National Centre of Competence in Research (NCCR) Catalysis, University of Geneva, Quai Ernest-Ansermet 30, 1211 Geneva 4, Switzerland. [c] Max Planck Institute for Marine Microbiology, Celsiusstraße 1, 28359 Bremen, Germany. [d] Department of Chemical Engineering, Stanford University, Stanford, California 94305, United States. [e] Department of Organic Chemistry, National Center of Competence in Research (NCCR) Chemical Biology, University of Geneva, Quai Ernest-Ansermet 30, 1211 Geneva 4, Switzerland.

Data availability:

All datasets are freely available on Zenodo: https://doi.org/10.5281/zenodo.6865680

Azotobacter vinelandii cultures

All buffers and solutions were prepared with MilliQ water (18.2 MΩ cm). Generally, large volumes (> 50 mL) were sterilized by autoclaving; smaller solutions were sterilized by filtration (0.45 μm syringe filters).

Agar culture medium

A modified Burke's medium was used for the cultivation of all *Azotobacter vinelandii* strains. Under sterile conditions, 6 mL of an autoclaved 100x phosphate buffer (0.46 M K_2HPO_4, 0.15 M KH_2PO_4) was added to an autoclaved molten agar solution (~18 g agar per L of water). 27.5 mL of this resulting agar buffer was added to 3 mL of an autoclaved 10x salt solution (60 mM sucrose, 8.1 mM $MgSO_4 \cdot 7H_2O$, 6.1 mM $CaCl_2$), 250 μL of a filter-sterilized 3 M NH_4OAc solution, 30 μL of a filter-sterilized 0.1 M Fe solution ($FeCl_3.6H_2O$) and 30 μL of a filter-sterilized 10 mM Mo solution ($Na_2MoO_4.2H_2O$). The mixture was poured in a Petri dish, solidified, and the relevant *A. vinelandii* strains were plated and incubated at 30 °C for 48 h.

Liquid culture medium

Under sterile conditions, 60 mL of an autoclaved 100x phosphate buffer solution (0.46 M K_2HPO_4, 0.15 M KH_2PO_4) was added to 6 L of autoclaved salt medium (60 mM sucrose, 8.1 mM $MgSO_4 \cdot 7H_2O$, 6.1 mM $CaCl_2$) after both solutions were at room temperature. Then 20 mL of a filter-sterilized 3 M NH_4OAc solution, 6 mL of a filter-sterilized 0.1 M Fe solution ($FeCl_3.6H_2O$) and 6 mL of a filter-sterilized 10 mM Mo solution ($Na_2MoO_4.2H_2O$) was added (yielding a modified Burke's medium).

Large scale cultures

For each *A. vinelandii* strain, a preculture was grown in a 250 mL baffled culture flask (glass) with a vented cap containing 60 mL of sterile liquid media. Each preculture was started by inoculation from a fresh agar plate and grown aerobically at 200 rpm at 30 °C until $OD_{600\ nm}$ > 2.0 (usually overnight). Under sterile conditions, 60 mL of the preculture was used to inoculate the 6 L of liquid media in 2x 5 L baffled flasks (Corning CLS431684, 3 L of culture per bottle) with vented caps. Cells were grown at 200 rpm at 30 °C overnight until $OD_{600\ nm}$ > 1.5 (usually overnight). Cells were collected by serial centrifugation runs at 4,500 x *g*, 20 min at room temperature. The supernatant was discarded, and the cells were gently resuspended in fresh growth medium devoid of NH_4OAc or another source of fixed nitrogen (prepared fresh, but not under sterile conditions) in the incubator shaker (~400 mL media per bottle). After resuspension, the culture was divided evenly between the remaining media/bottles and incubated at 200 rpm and 30 °C for another 3 hours to facilitate derepression of the *nif* operon, and harvested by serial centrifugation collections as above. Finally, the cells were scooped into a plastic bag and stored at –80 °C until further use.

Azotobacter vinelandii strains

"Wild-type" MoFe protein contains an N-terminal poly(histidine)$_8$ tag between codons 2 and 3 of *nifD*. The strain containing this construct was prepared previously by markerless mutagenesis (yielding *A. vinelandii* RS1, derived from strain DJ).[1,2] α-C45A/L158C MoFe protein was prepared from this strain (detailed below), and therefore also carries a poly(histidine)$_8$ tag between codons 2 and 3 of *nifD*.[2] L127Δ Fe protein was purified from *A. vinelandii* strain DJ1065, which was generously shared by Dennis Dean (Virginia Tech). A Strep-tag-containing MoFe protein (N-terminal of NifD) was purified from *A. vinelandii* strain DJ2102, which was generously shared by Dennis Dean (Virginia Tech); this protein was used as a control only in western blotting experiments.

Preparation of A. vinelandii strain producing α-C45A/L158C MoFe protein

All *A. vinelandii* strains were maintained in a modified Burke's medium. When required, kanamycin was added at a final concentration of 3 μg/mL (*A. vinelandii*). *Escherichia coli* DH5α was cultivated in Luria-Bertani medium at 37 °C. When required, kanamycin was added to 25 μg/mL (*E. coli*).

Plasmid constructions were performed using *E. coli* DH5α (Invitrogen, USA). A *sacB*-based system was used to create the α-C45A/L158C double-mutation in *A. vinelandii via* double homologous recombination.[3,4] During this process, *A. vinelandii* cells were maintained in Burke's medium with glucose as the substrate. Briefly, pK18*mobsacB* based vectors were transformed into *A. vinelandii* as described before.[1,2] We thank Professor Jeremy D. Semrau at University of Michigan (US) for providing plasmid pK18*mobsacB*. Successful transformants were kanamycin-resistant, suggesting successful single homologous recombination and incorporation of the plasmid, and transferred at least one more time on kanamycin-plates before counter selection. The transformants were then grown in liquid medium without antibiotics for a few hours to overnight for outgrowth. Various amounts of cells were then spread on agar plates with glucose as the substrate and with addition of 3% sucrose for counter selection of the second homologous recombination. Sucrose-resistant colonies were transferred at least one more time on 3% sucrose selection plates before further phenotype screening. Desired clones were sucrose-resistant and kanamycin-sensitive suggesting loss of pK18*mobsacB* backbone and were first unable of diazotrophic growth (for *A. vinelandii* Δ*nifD*) and had restored diazotrophic growth ability for the *A. vinelandii nifD*:C45A/L158C mutant. Mutations were also confirmed by PCR and Sanger sequencing.

To create the *A. vinelandii nifD*:C45A/L158C mutant, an *A. vinelandii* Δ*nifD* mutant was first constructed with the in-frame deletion from 45C to 158L (Figure S1) in strain *A. vinelandii* RS1. Briefly, two flanking regions were amplified using primer sets del*nifD*_F1/R1 and del*nifD*_F2/R2 (Table S1) and inserted into the suicidal vector pK18*mobsacB* using NEBuilder® HiFi DNA Assembly (New England Biolabs, US) to create vector pK18del*nifD* (Figure S1). The pK18*mobsacB* plasmid was linearized *via* digestion by HindIII and BamHI enzymes (New England Biolabs, US). This vector was used to create a Δ*nifD* mutant in *A. vinelandii* RS1 *via* double homologous recombination.

Subsequently, a second mutation vector was constructed by re-inserting the deleted *nifD* region carrying the desired point mutations (Figure S1). Briefly, vector pK18del*nifD* was linearized by HindIII restriction digestion previously included in del*nifD*_R1 and del*nifD*_F2 primers (Table S1). Partial *nifD* was amplified using primers pm*nifD*_InsF/R with point mutations introduced on both primers (Table S1). This fragment was inserted into pK18del*nifD via* Gibson assembly to create vector pK18del*nifD*:C45A/L158C (Figure S1). This vector was transformed into *A. vinelandii* Δ*nifD* for double homologous recombination, restoration of diazotrophic growth, and introduction of the desired point mutations. *Note:* The wild-type (non-His-tag-containing) numbering is used here. Including the poly(histidine)$_8$ tag, α-C45A/L158C becomes α-C53A/L166C.

Table S1: Primers, vectors, and strains used in this study.

**Primers**	
(underlined regions are overhangs for Gibson assembly, ***bolded capital*** *letters are point mutations, sequences in parentheses are HindIII restriction sites inserted for linearizing plasmid)*	
del*nifD*_F1	cgttgtaaaacgacggccagtgccatcgtattctacgacgtgctgggcg
del*nifD*_R1	cacggattcgatgtcgtcgccgat(aagctt)cttcttggactgggtaaccgccg
del*nifD*_F2	cccggcggttacccagtccaagaag(aagctt)atcggcgacgacatcgaatccg
del*nifD*_R2	cgaattcgagctcggtacccgggcggagccgatcaggtcgggctt
pm*nifD*_InsF	cggttacccagtccaagaag**GC**catcatctccaacaagaagtcccagcc
pm*nifD*_InsR	ggattcgatgtcgtcgccgat**GCA**gccgatcgggcactcggactg
Av-genome-sequencing_1	aacaacatctccaagggcatcg
Av-genome-sequencing_2	gtatcaaggagaagttcatcttcca
Av-genome-sequencing_3	gcagtttcttccagcacggatt
Av-genome-sequencing_4	tttccgactccatgaccgaa
Av-genome-sequencing_5	ggtatcggctcgatgaaataggtc
**Vectors**	
pK18*mobsacB*	Km^r RP4-mob, mobilizable cloning vector containing *sacB* from *Bacillus subtilis*.[5]
pK18del*nifD*	pK18*mobsacB* carrying flanking regions to knock out *nifD*.
pK18del*nifD*: C45A/L158C	pK18*mobsacB* carrying flanking regions to create point mutations in *nifD*: C45A/L158C.
**Strains**	
E. coli DH5α	F^- φ80*lac*ZΔM15 Δ(*lac*ZYA-*arg*F)U169 *rec*A1 *end*A1 *hsd*R17(r_K^-, m_K^+) *pho*A *sup*E44 λ^-*thi*-1 *gyr*A96 *rel*A1 (Invitrogen, USA)
A. vinelandii RS1	8xHIS-tag between codons 2/3 of *nifD*.[1,2] Comparable to strain DJ1141.[6] Produced from *A. vinelandii* DJ (BTCC).
A. vinelandii Δ*nifD*	In-frame deletion of *nifD* from 45C to 158L from *A. vinelandii* RS1.
A. vinelandii nifD: C45A/L158C	Point mutations of *nifD*:C45A/L158C. Analogous to MoFe protein in Ref[2]
A. vinelandii DJ1065	Strain of *A. vinelandii* producing NifH with residue 127L deleted (Dennis Dean).[7,8]
A. vinelandii DJ2102	Strain of *A. vinelandii* producing MoFe protein with an Nterm Strep-tag on NifD (Dennis Dean).[9]

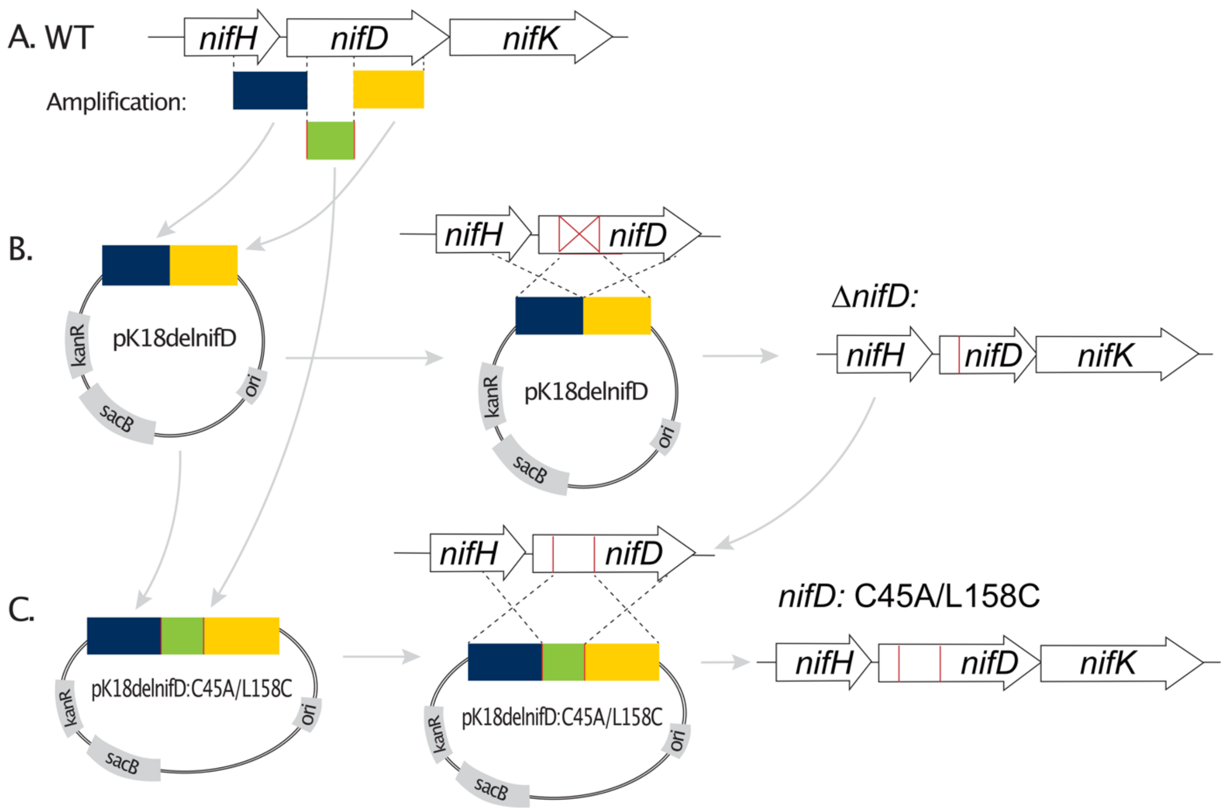

Figure S1: Graphical scheme of deletion of *nifD* in *A. vinelandii* (steps A and B), and the point mutations of C45A/L158C in *nifD* (steps A and C). Red lines across *nifD* represents either deletion or point mutation.

***Sequence of nifD encoding α-C45A/L158C* NifD**

nifD in red

nifK in orange

gcc = α-C45A single-point mutation

tgc = α-L158C single-point mutation

caccaccatcatcaccaccatcac = poly(histidine)$_8$ tag

Forward sequencing primers are highlighted in yellow

Reverse sequencing primers are highlighted in cyan

Regions unconfirmed by sequencing are highlighted in grey.

aacaacatctccaagggcatcgtgaagtatgccaactccggcagcgtgcgtctgggcggcctgatctgcaacagccgtaacaccgaccgcgaag
acgagctgatcatcgctctggccaacaagctgggcacccagatgatccacttcgtgccgcgtgacaacgtcgtgcagcgcgccgaaatccgccg
catgaccgtgatcgaatacgatccgaaagccaagcaagccgacgaataccgcgctctggcccgcaaggtcgtcgacaacaaactgctggtcatc
ccgaacccgatcaccatggacgagctcgaagagctgctgatggaattcggcatcatggaagtcgaagacgaatccatcgtcggcaaaaccgcc
gaagaagtctgatagccgctccggtttcagaaggacgggacagggcagattggctctgtcggggtggcgccccccgcatggggcgggcgcccc
acccgttacccgcatatgaacgctaaggcaagaggagtcatacccatgacccaccaccatcatcaccaccatcacggtatgtcgcgcgaagagg
ttgaatccctcatccaggaagttctggaagtttatcccgagaaggctcgcaaggatcgtaacaagcacctggccgtcaacgacccggcggttacc
cagtccaagaag**gcc**atcatctccaacaagaagtcccagcccggtctgatgaccatccgcggctgcgcctacgccggttccaaaggcgtggtct

ggggccccatcaaggacatgatccacatctcccacggtccggtaggctgcggccagtattcgcgcgccggccgtcgtaactactacatcggtacc
accggtgtgaacgccttcgtcaccatgaacttcacctcggacttccaggagaaggacatcgtgttcggtggcgacaagaagctcgccaaactgat
cgacgaagtggaaaccctgttcccgctgaacaagggtatctccgtccagtccgagtgcccgatcggc**tgc**atcggcgacgacatcgaatccgtgt
ccaaggtcaagggcgccgagctcagcaagaccatcgtaccggtccgttgcgaaggcttccgcggcgtttcccagtccctgggccaccacatcgc
caacgacgcagtccgcgactgggtcctgggcaagcgtgacgaagacaccaccttcgccagcactccttacgatgtggccatcatcggcgactac
aacatcggcggcgacgcctggtcttcccgcatcctgctggaagaaatgggcctgcgttgcgtagcccagtggtccggcgacggctccatctccga
aatcgagctgaccccgaaggtcaagctgaacctggttcactgctaccgctcgatgaactacatctcccgtcacatggaagagaagtacggtatcc
catggatggagtacaacttcttcggcccgaccaagaccatcgagtcgctgcgtgccatcgccgccaagttcgacgagagcatccagaagaagtg
cgaagaggtcatcgccaagtacaagcccgagtgggaagcggtggtcgccaagtaccgtccgcgcctggaaggcaagcgcgtcatgctctacat
cggtggcctgcgtccgcgccacgtgatcggcgcctacgaagacctgggcatggaagtggtgggtaccggctacgagttcgcccacaacgacga
ctatgaccgcaccatgaaagaaatgggtgactccaccctgctgtacgatgacgtgaccggctacgaattcgaagaattcgtcaagcgcatcaag
cccgacctgatcggctccggtatcaaggagaagttcatcttccagaagatgggcatccccttccgtcaaatgcactcctgggattattccggcccct
accacggcttcgatggcttcgccatcttcgcccgtgacatggacatgaccctgaacaatccgtgctggaagaaactgcaggctccctgggaagct
tccgaaggcgccgagaaagtcgccgccagcgcctgatagcagagcaatcgtacgcaacgtccgctgcgggcggtttccgcccgcggccgacat
ccgctaacgccgttcacagatgagtgaggcgtaggagagagtcatgagccagcaagtcgataaaatcaaagccagctacccgctgttcctcgat
caggactacaaggacatgcttgccaagaagcgcgacggcttcgaggaaaagtatccgcaggacaagatcgacgaagtattccagtggaccacc
accaaggaataccaggagctgaacttccagcgcgaagccctgaccgtcaacccggccaaggcttgccagccgctgggcgccgttctctgcgccc
tcggtttcgagaagaccatgccctacgtgcacggttcccagggttgcgtcgcctacttccgctcctacttcaaccgtcatttccgcgagccggtttcc
tgcgtttccgactccatgaccgaagacgcggcagtgttcggcggccagcagaacatgaaggacggtctgcagaactgtaaggctacctacaagc
ccgacatgatcgcagtgtccaccacctgcatggccgaggtcatcggtgacgacctcaacgccttcatcaacaactcgaagaaggaaggtttcatt
cctgacgagttcccggtgccgttcgcccataccccgagcttcgtgggcagccacgtgaccggctgggacaacatgttcgaaggcattgctcgcta
cttcaccctgaagtccatggacgacaaggtggttggcagcaacaagaagatcaacatcgtccccggcttcgagacctacctgggcaacttccgc
gtgatcaagcgcatgctttcggaaatgggcgtgggctacagcctgctctccgatccggaagaagtgctggacaccccggctgacggccagttccg
catgtacgcgggcggcaccactcaggaagagatgaaggacgctccgaacgccctcaacaccgtcctgctgcagccgtggcacctggagaagac
caagaagttcgtcgagggtacctggaagcacgaagtaccgaagctgaacatcccgatgggcctggactggaccgacgagttcctgatgaaagt
cagcgaaatcagcggccagccgattccggcgagcctgaccaaggagcgtggccgtctggtcgacatgatgaccgactcccacacctggctgca
cggcaagcgtttcgccctgtggggtgatccggacttcgtgatgggcctggtcaagttcctgctggaactgggttgcgagccggtacacattctctg
ccacaacggcaacaagcgttggaagaaggcggtcgacgccatcctcgccgcttcgccctacggcaagaatgctaccgtctacatcggcaagga
cctgtggcacctgcgttcgctggtcttcaccgacaagccggacttcatgatcggcaacagctacggtaagttcatccagcgcgacaccctgcaca
agggcaaggagttcgaggttccgctgatccgtatcggcttcccgatcttcgaccgtcatcacctgcatcgctccaccaccctgggttacgagggcg
ccatgcagatcctgaccaccctggtgaactcgatcctggaacgtctggacgaggaaacccgcggtatgcaggccaccgactacaaccacgacct
ggtacgctaagtcgtcggttcaagtggtatcggccggagcggcgcaagccgctccggctcttccttggcggcggccgcaggtggtcgggcctttt
gcccgcgatctgcggcaaccgccaaacccgtctaaggagcaagcccatgcccagcgtcatgattcgccgcaacgacgaaggccaactgaccttc
tatatcgccaagaaagaccaggaagagatcgtggtgtccctggagcatgacagccccgaactctggggtggcgaagtcaccctcggcgacggtt
cgacctatttcatcgagccgatacc

Purification of MoFe and Fe proteins:

All buffers were prepared with MilliQ water and deoxygenated inside of a COY glovebox (>95% N_2/<5% H_2, Coy Laboratory Products, USA) before use (at least overnight). All subsequent procedures were conducted within the glovebox, with the exception of centrifugation steps (performed in deoxygenated and anoxically sealed poly(propylene) bottles).

Resuspension and lysis

Cell pellets were anaerobically thawed and resuspended for 20 minutes in lysis buffer (50 mM Tris/HCl pH 8, containing 5 mM dithionite (abbreviated as DT), and 37% v/v glycerol) with a ratio 2:1 (buffer:cells v/w), and subsequently collected by centrifugation at 12,000 x *g* (20 min at 4 °C). Once again inside of the glovebox, the glycerol-containing supernatant was discarded, and cells were rapidly lysed by osmotic shock/shaking following the introduction of an equal replacement volume of glycerol-free lysis buffer (in presence of few µg of DNase). After incubation on ice for approximately 15 minutes, the cell lysate was clarified by centrifugation at 30,000 x *g* (4 °C, 1 hour) to yield a dark brown supernatant.

Purification of MoFe (WT and α-C45A/L158C) proteins

Note: From here onwards, DT was omitted from all buffers.

Within the glovebox, the supernatant was decanted and diluted with a post-lysis buffer (2 M NaCl, 234 mM Tris/HCl, pH 8.0) in order to obtain a final NaCl concentration of 0.3 M NaCl. His-tagged "wild-type" and α-C45A/L158C MoFe protein were purified by Ni-affinity chromatography using a Ni-loaded HisTrap HP 5 mL column (Cytiva) with a flow rate of 5 mL/min using an Äkta Start system within the glovebox. The MoFe-containing supernatant was loaded onto the column, washed with an equilibration buffer for at least 3 column volumes to remove remaining DT (0.3 M NaCl, 50 mM Tris/HCl, pH 8.0), washed with a 20 mM imidazole step, and finally eluted with a 0.3 M imidazole step (0.3 M imidazole, 0.3 M NaCl, 50 mM Tris/HCl, pH 8.0). The eluted MoFe protein was then purified further with a HiPrep Q-Sepharose FF 16/10 column (Cytiva, 20 mL column volume, flow rate = 5 mL/min), which also served to remove excess imidazole and residual DT. The concentration of NaCl in the MoFe protein samples was first lowered to 0.1 M NaCl using a dilution buffer (50 mM Tris/HCl, pH 8.0) before being loaded on the pre-equilibrated Q-sepharose column (0.1 M NaCl). MoFe proteins were eluted at approximately 0.3 M NaCl using a NaCl gradient over 7 column volumes (from 0.2 to 0.65 M NaCl). MoFe proteins were typically concentrated to >20 mg/mL using a Merck Millipore stirred concentrator cell (within the glovebox) equipped with a 100 kDa molecular weight cut-off membrane (fed with ultra-high-purity N_2 5.0). MoFe proteins were subsequently flash-frozen as 20 µL pellets in liquid nitrogen and stored in liquid nitrogen until further use. Protein concentrations were determined by the Biuret method using bovine serum albumin as the standard.

Purification of Fe (WT and L127Δ) proteins

In contrast to the MoFe proteins, Fe protein samples were purified in the presence of 2 mM DT in all buffers (5 mM DT was included in the cell lysis step, as above). Fe proteins were routinely purified in tandem to the MoFe proteins using an Äkta Go system. Following cell lysis (L127Δ Fe protein) the supernatant was diluted with a post lysis buffer (2 M NaCl, 234 mM Tris/HCl, 2 mM DT, pH 8.0) in order to obtain NaCl concentration of 0.3 M NaCl. WT Fe proteins were purified from His-column flow-through fractions (above), where the flow-through fraction was first diluted to 0.1 M NaCl using the dilution buffer above (containing 2 mM DT). The Fe protein was first purified using a HiPrep Q-Sepharose HP 16/10 column with a flow rate of 5 mL/min. Fe protein elution was performed using a NaCl gradient (7 column volumes, from 0.2 to 0.65 M NaCl) and concentrated to <10 mL using a Merck-Millipore stirred concentrator cell (30 kDa molecular weight cut-off membrane). The Fe protein was then purified further by size-exclusion chromatography (HiPrep 26/60 Sephacryl S-200 HR, Cytiva) with a flow rate of 2.5 mL/min, using a Tris running buffer (50 mM Tris/HCl, pH 8.0, 0.5

M NaCl). Eluted Fe protein samples were concentrated to >20 mg/mL, flash frozen in liquid nitrogen as 20 μL pellets and stored in liquid nitrogen until further use. Protein concentrations were determined by the Biuret method using bovine serum albumin as the standard.

Functionalization with maleimide-desthiobiotin ("DTB") inhibitor
DT-free MoFe protein was reacted in the presence of increasing molar equivalents of DTB-inhibitor (synthesis below, fresh stocks prepared at 1 – 3 mg/mL within the glovebox using 0.1 M MOPS/NaOH buffer, pH 7) for 4 hours at room temperature within the glovebox. Time reactions were quenched by the addition of 2 mM DT (final concentration) to the reaction to rapidly reduce the maleimide functional group. Activity assays were performed immediately (see below).

A control reaction was performed in which 26 μM DTB-maleimide inhibitor (confirmed by mass spectrometry) was incubated in 2 mM DT-containing MOPS/NaOH buffer (0.1 M, pH 7) for 30 minutes at room temperature. Upon analysis of the reaction mixture was analyzed by LC-MS after this time the starting material was no longer visible, consistent with the reduction of the maleimide by DT (consistent with the results presented in Figure S8).

As discussed in the main article, we attempted to purify the DTB-conjugated MoFe proteins using commercial Streptactin-based solid phases (specifically, "StrepTrap" by Cytiva). After failing to observe the association of DTB-modified MoFe proteins to this solid phase, we deemed it prohibitively costly to employ an alternative Streptavidin-containing stationary phase; Streptavidin cannot be efficiently regenerated following incubation with biotin (to elute the DTB-functionalized protein).

Functionalization with Strep-tag inhibitor and conjugate purification
A synthetic peptide with the sequence GGGWSHPQFEK and containing an N-terminal maleimide functional group was synthesized by Genscript (USA). DT-free MoFe protein was reacted with 0.5 molar equivalents (per MoFe protein) of Strep-maleimide inhibitor to minimize the formation of di-functionalized MoFe proteins for 4 hours at room temperature within the glovebox (additional information with respect to these molar ratios is presented below, Figure S10). Stock solutions of the Strep-maleimide peptide were prepared fresh within the glovebox using 0.1 M MOPS/NaOH buffer (pH 7), with concentrations between 1 – 3 mg/mL. Functionalization reactions were quenched by the addition of DT (2 mM final concentration). The Strep-inhibited (Strep-functionalized) α-C45A/L158C MoFe protein was subsequently purified from unreacted protein using a StrepTrap-XT 1 mL column (Cytiva) equilibrated with MOPS buffer (0.1 M MOPS/NaOH, 0.2 M NaCl, 2 mM DT, pH 7.0). Unreacted MoFe was collected as the flow-through (FT) fraction. Strep-functionalized protein was subsequently eluted in a single step with biotin-containing buffer (50 mM biotin, 0.1 M MOPS/NaOH, 0.2 M NaCl, 2 mM DT, pH 7.0). Samples were concentrated separately with a Merck-Millipore stirred concentrator cell equipped with a 100 kDa molecular-weight cut-off membrane. Protein samples were flash frozen in liquid nitrogen as 20 μL pellets and stored in liquid nitrogen until further use.

Activity assays
Reduction activity assays were conducted in triplicate 1 mL reactions in 13 mL septum-sealed glass vials (Wheaton) containing deoxygenated buffer and an ATP-regenerating system (5 mM ATP, 30 mM phosphocreatine, 1.3 mg BSA, 0.2 mg creatine phosphokinase (from Rabbit muscle), 10 mM sodium dithionite and 100 mM MOPS/NaOH at pH 7.0). All reactions contained 0.1 mg MoFe protein and 16.6 molar equivalents of Fe protein (0.48 mg). All reaction vials were sealed within an Ar-filled glovebox (Jacomex, France) and vented to atmospheric pressure. Where necessary, vials were flushed with ultra-high-purity N_2 (or desired quantities were introduced using gas-tight syringes); all vials were

vented to atmospheric pressure before reactions were started. Reactions were performed within a shaking water bath (30 °C) and initiated by the addition of $MgCl_2$ (from a 1 M stock, 10 mM final concentration) using a gas-tight syringe. Reactions were quenched after 8 minutes by the addition of 300 µL of 400 mM EDTA (pH 8.0). H_2 quantification was performed on all reactions using a calibrated GC-TCD equipped with a molecular sieve 5 Å column (Ar carrier, SRI Instruments model 8610C). Ammonia was quantified by the *ortho*-phthalaldehyde method (corrected to controls and assays performed under 1 atm Ar) using NH_4Cl as the standard, as reported previously.[1,10]

Analytical Gel filtration

Analytical gel filtration was performed using a Superose 6 Increase 10/300 GL column (Cytiva), using an Äkta Start within an anoxic glovebox. The proteins were diluted with the running buffer (50 mM Tris/HCl, 500 mM NaCl, pH 8, 0.5 mM DT) to a concentration of 20 µmol/mL for MoFe protein and 100 µmol/mL for L127Δ Fe protein. 200 µL aliquots were prepared and incubated for one hour before being loaded (100 µL) onto the pre-equilibrated column.

Limited Proteolysis

A 50 mM Tris/HCl (pH 7.6) buffer containing 10 mM $CaCl_2$ buffer was prepared and deoxygenated in a Ar-filled glovebox (Jacomex, France) overnight. The buffer was split in 2 bottles and 0.2 mg/mL fresh trypsin (Sigma) was added to the first. This trypsin-containing buffer was then activated at 30 °C for 20 minutes using a heating block. Proteins were brought in the glovebox and diluted to a concentration of 1 mg/mL using the buffer that did not contain trypsin. The proteins were placed in LC/MS vials with stirring in a water bath at 37 °C and the activated trypsin was then added to the protein samples to a final concentration of 2 µg/mL (trypsin, 500:1 MoFe:trypsin) as the starting point for the reaction.

The protein reactions were sampled at time 0 (before trypsin addition), 2, 5, 30, 60 and 90 minutes after trypsin addition. 4 µL of samples of the reactions was transferred to Eppendorf tubes containing that already contained 4 µL TruPAGE™ LDS (4X) sample buffer (Merck, Switzerland), 1.6 µL 1 M dithiothreitol (DTT) and 6.4 µL MilliQ water. The tube was then immediately placed at 95 °C on a heating block for 5 minutes to deactivate the trypsin and terminate proteolysis. 4-12% mPAGE® Bis-tris gels (Merck, Switzerland) were used for analysis. 7 µL aliquots of the treated protein samples were loaded in each well. The gel was run at 200 V and stained with One-Step Blue® (Biotium, Brunschwig Switzerland).

Native PAGE

All the reagents and equipment were purchased from Invitrogen™/ThermoFisher scientific (Switzerland). The buffers were prepared using NativePAGE™ Running Buffer (20X) and NativePAGE™ Cathode Buffer Additive (20X). Buffers were diluted in an N_2-filled glovebox with anoxic MilliQ water. 0.5 mM DT was added to the cathode buffer. The gel (NativePAGE™ 3 to 12%, Bis-Tris) was placed in the cassette which was then filled with the two separate buffers. The gel was then run for 5 minutes prior to sample loading at 150 V. The protein samples were then diluted to a concentration of 0.2 mg/mL with 50 mM Tris (containing 0.5 mM DT) in the glovebox. 2.5 µL of NativePAGE™ Sample Buffer (4X) was then added to 7.5 µL of protein, and then 10 µL of the resulting samples (and 5 µL of protein ladder, NativeMark™ unstained protein standard) were loaded on the gel which was subsequently run in the glovebox at 150 V. The gel was stained with Coomassie following the protocol from Invitrogen™.

Western blotting

Protein samples (0.5 - 1 µg per well) were loaded on a 4-15% TGX Stainfree gel (BioRad, Switzerland) and run for 40 min at 220 V. The gel was irradiated with UV light for 1 min to activate the Stainfree

component, before transferring the protein to PVDF membranes using the Trans-Blot Turbo Transfer System (Bio-Rad). The membranes were scanned for total protein (Stainfree), before being blocked with 10% Sea Block blocking buffer (ThermoFisher Scientific, Switzerland) in PBS for 1h at room temperature. Membranes were incubated overnight with 1:10000 HRP-Streptavidin (BioLegend, Lucerna-Chem Switzerland) or 1:10000 HRP-Strep-tactin (IBA lifesciences, Lucerna-Chem Switzerland) in 10% Sea Block/TBST (TRIS/HCl-buffered (20 mM pH 7.4-7.6) saline (150 mM) with 0.1% tween-20) at 4 °C, washed with TBST (4x 5 min), and the antibodies detected by chemiluminescence using Supersignal West Pico Plus chemiluminescent substrate and a Fusion imager (Vilber).

Proteomics analysis and specificity of the maleimide-Cys reaction
Raw proteomics data and detailed proteomics reports provided by the Proteomics Core Facility within the Faculty of Medicine at the University of Geneva have been uploaded to Zenodo (link on the first page). Briefly, Strep-inhibited proteins were purified over a StrepTrap XT (Cytiva Switzerland, Streptactin solid phase) to separate unmodified proteins. Next, these proteins were run into an SDS-PAGE gel, the band excised and stored in 10% acetic acid, and sent to the core proteomics facility for analysis. This was performed for Strep-inhibited α-C45A/L158C MoFe protein and for wild-type MoFe protein; the wild-type MoFe protein was prepared as above for the α-C45A/L158C MoFe protein, although 5 molar equivalents of Strep-maleimide were used per MoFe protein. After in-gel digestion with trypsin, samples were analyzed by LC-ESI-MS/MS using a Q-Exactive HF Hybrid Quadrupole-Orbitrap Mass Spectrometer (ThermoFisher Scientific) equipped with an Easy nLC 1000 Liquid Chromatography system (ThermoFisher Scientific) for modified cysteine residues. A peaklist was generated using ProteoWizard and searched against the Uniprot database for *A. vinelandii* DJ, with the addition of a modified version of *nifD* due to the expected α-C45A/L158C mutation. A subsequent search was also performed in order to identify potential Strep-inhibitor modifications on any amino acids. A Mascot search of the results was analyzed using Scaffold 5.0.0 (Proteome software); identified peptides were accepted only in the case of having a Percolator posterior error probability of >95%.

Under these conditions, the Strep-maleimide modification was detected on the expected trypsin-digested peptide of NifD, GISVQSE<u>C</u>PIG**C**IGDDIESVSK, where the bold Cys represents the target α-C158 residue and the underlined Cys represents a buried P cluster-coordinating α-C154 residue. Further, both unmodified and modified version of this peptide fragment were identified, consistent with the partial functionalization of $\alpha_2\beta_2$ α-C45A/L158C MoFe protein. The subsequent broader search for the potential modification of the Strep-inhibitor on any amino acid identified three low-quality hits for a single partially solvent-exposed peptide fragment on the ß-subunit (NifK).

In order to clarify the selectivity of the Strep-inhibitor maleimide for our targeted cysteine residue, analyses were repeated using wild-type MoFe protein functionalized with an excess (5 molar equivalents) of the Strep-maleimide inhibitor (StrepTactin affinity purified as above). A Strep-modified GISVQSE<u>C</u>PIG**L**IGDDIESVSK peptide was not detected (the unmodified peptide was detected), consistent with the Strep-maleimide inhibitor's selectivity towards the target α-C158 residue. Neither an unmodified nor a Strep-modified peptide fragment containing the original surface-exposed α-C45 residue was detected in this sample (although the sample was purified over the StrepTrap XT column, as above); this was hypothesized to be due to the small size of the target peptide (outside of the MS detection window). Importantly, the potential ß-subunit (NifK) modification identified above was not identified in the presence of excess Strep-inhibitor, consistent with the above identification indeed being an experimental artefact.

Two additional database searches were performed on the raw data by Dr Yibo Wu within the Department of Organic Chemistry at the University of Geneva. Interestingly, the search engine SpectroMine did not return a Strep-functionalized peptide fragment for the α-C45A/L158C MoFe protein. An additional search on the Proteome Discover database (v2.4) corroborated the desired functionalization of the α-C158 residue, although an additional modification was identified on the FeMoco-coordinating α-C275 residue. Since (i) the α-C45A/L158C MoFe protein has high specific activity values (this work and in Ref[2]), (ii) X-ray crystallography confirms the double occupancy of the MoFe protein with FeMoco and (iii) a Strep-functionality at this buried residue is unlikely (and would not permit purification over the StrepTrap XT column), this is anticipated to be a minor artefact due to reversible maleimide-Cys degradation during peptide digestion/sample preparation.[11]

An additional control experiment was performed using an additional α-C45S MoFe protein mutant (purified from *A. vinelandii* strain DJ2192, producing a MoFe protein with a His-tag on the N-term). The Strep-inhibition protocol outlined above was repeated and the reaction product was purified as above. Importantly, the reaction efficiency decreased from 14% (for the α-C45A/L158C MoFe mutant) to 7% (for the α-C45S MoFe mutant), indicating that the maleimide-Cys may not be entirely selective. We hypothesize that this promiscuous reactivity could have been further enhanced in the absence of a solvent exposed Cys residue on this mutant. Additional analysis of this α-C45S MoFe mutant (Figures S18-20) also indicated that this non-specific Strep-inhibitor modification may also inhibit Fe protein access to the MoFe protein interaction site. This is easily explained by the relatively large size and potential flexibility of the Strep inhibitor.

Table S2 X-ray analysis statistics.

**Data collection**	
Wavelength (Å)	1.00003
Space group	$P2_1$
Resolution (Å)	143.49 – 3.03 (3.49 – 3.03)
Cell dimensions	
a, b, c (Å)	148.39, 73.44, 211.25
α, β, γ (°)	90.00, 104.76, 90.00
R_{merge}(%)[a]	29.7 (51.4)
R_{pim} (%)[a]	17.5 (33.4)
$CC_{1/2}$ [a]	0.957 (0.806)
I/σ_I [a]	3.4 (1.6)
Spherical completeness[a]	46.2 (6.8)
Ellipsoidal completeness[a]	86.6 (69.4)
Redundancy[a]	3.7 (3.3)
Nr. unique reflections[a]	39,936 (1,998)
**Refinement**	
Resolution (Å)	95.50 – 3.03
Number of reflections	39,924
R_{work}/R_{free} [b] (%)	20.37/22.27
Number of atoms	
Protein	31,844
Ligands/ions	314
Solvent	0
Mean B-value (Å^2)	35.11
Molprobity clash score, all atoms	2.14
Ramachandran plot	
Favored regions (%)	96.58
Outlier regions (%)	0.15
rmsd[c] bond lengths (Å)	0.004
rmsd[c] bond angles (°)	0.664
**PDB ID code**	**8BTS**

[a] Values relative to the highest resolution shell are within parentheses. [b] R_{free} was calculated as the R_{work} for 5 % of the reflections that were not included in the refinement. [c] rmsd, root mean square deviation.

Table S3: Comparison of the unit cell dimensions between the crystal structures of MoFe proteins.

Entity ID	Length a (Å)	Length b (Å)	Length c (Å)	Angle α (°)	Angle β (°)	Angle γ (°)	Molecules/AU	Space Group
5BVG	77.582	130.825	107.128	90	108.85	90	4	*P* 1 21 1
5BVH	81.071	130.833	107.318	90	110.64	90	4	*P* 1 21 1
4ND8	76.527	127.915	107.082	90	108.9	90	4	*P* 1 21 1
4TKU	80.941	130.785	107.005	90	110.58	90	4	*P* 1 21 1
4TKV	81.172	130.623	106.996	90	110.65	90	4	*P* 1 21 1
1G20	264.24	111.46	121.59	90	97.4	90	8	*C* 1 2 1
1G21	110.5	121.5	264.9	90	90	90	8	*P* 21 21 21
6CDK	107.88	130.78	80.789	90	110.85	90	4	*P* 1 21 1
6UG0	84.362	156.967	202.343	90	90	90	4	*P* 21 21 2
5VQ4	81.315	128.859	108.359	90	110.89	90	4	*P* 1 21 1
4WNA	77.123	129.78	107.543	90	108.94	90	4	*P* 1 21 1
3U7Q	81.19	130.696	107.224	90	110.67	90	4	*P* 1 21 1
6O7R	76.61	128.81	107.64	90	109.07	90	4	*P* 1 21 1
6O7S	76.775	128.05	107.539	90	108.97	90	4	*P* 1 21 1
6O7P	76.847	128.439	107.707	90	109.06	90	4	*P* 1 21 1
6O7Q	77.437	130.413	107.751	90	109.09	90	4	*P* 1 21 1
6O7N	76.337	127.96	107.509	90	109.01	90	4	*P* 1 21 1
6O7O	76.746	128.678	107.536	90	108.94	90	4	*P* 1 21 1
6O7L	80.562	131.029	107.616	90	110.85	90	4	*P* 1 21 1
6O7M	76.457	127.788	107.539	90	109	90	4	*P* 1 21 1
7JRF	77.052	129.974	107.223	90	109.11	90	4	*P* 1 21 1
6VXT	163.535	203.435	83.857	90	103.945	90	4	*C* 1 2 1
1M34	326.1	75.8	312.2	90	102.6	90	16	*C* 1 2 1
3MIN	108	131.3	81	90	110.7	90	4	*P* 1 21 1
6BBL	76.781	128.251	107.272	90	109.11	90	4	*P* 1 21 1
1M1N	108.31	131.63	159.159	90	108.37	90	8	*P* 1 21 1
1L5H	150.452	191.922	102.339	90	90	90	2	*C* 2 2 21
2AFH	170.911	75.893	223.665	90	90	90	6	*P* 21 21 21
1M1Y	113.267	214.937	320.466	90	90	90	16	*P* 21 21 21
2MIN	107.7	130.2	81.3	90	110.8	90	4	*P* 1 21 1
2AFI	72.915	141.432	165.549	73.69	79.37	76.58	16	*P* 1
1N2C	79	299.7	334.5	90	90	90	8	*C* 2 2 21
3K1A	77.019	129.458	107.088	90	109.01	90	4	*P* 1 21 1
1FP4	107.2	130.2	80.4	90	111.2	90	4	*P* 1 21 1
4XPI	80.804	130.83	108.11	90	111.14	90	4	*P* 1 21 1
5CX1	175.502	144.595	177.747	90	114.27	90	16	*P* 1 21 1
4WZA	110.201	120.412	264.318	90	90	90	8	*P* 21 21 21
4WZB	110.533	120.894	264.834	90	90	90	8	*P* 21 21 21
6OP4	76.981	129.326	106.755	90	108.84	90	4	*P* 1 21 1
6OP3	80.872	131.023	106.957	90	110.62	90	4	*P* 1 21 1
6OP2	80.761	130.38	106.896	90	110.54	90	4	*P* 1 21 1
6OP1	77.191	128.003	107.633	90	109.15	90	4	*P* 1 21 1
αC45A αL158C	**148.392**	**73.444**	**211.253**	**90**	**104.76**	**90**	**8**	***P* 1 21 1**

MoFe protein crystalline form 1

MoFe protein other crystalline forms

MoFe protein crystalline form 2

Fe:MoFe complexes

Table S4: Structural alignment between the α-C45A L158C and MoFe-protein crystal structures. Models with high similarity (root mean square deviation, RMSD < 0.205 Å) to the α-C45A L158C model are bolded.

PDB code	Structure name	RMSD Å and (Superposed Cα)	PDB code	Structure name	RMSD Å and (Superposed Cα)
1FP4	Crystal structure of the alpha-H195Q mutant of nitrogenase	0.258 (1896)	5BVH	CO-bound form of Selenium incorporated nitrogenase MoFe-protein (Av1-Se-CO) from *A. vinelandii*	0.268 (1900)
1G20	Nucleotide-free structure of a nitrogenase protein complex between Leu127del-Fe protein and the MoFe protein	0.228 (1851)	**5CX1**	Nitrogenase molybdenum-iron protein beta-K400E mutant	**0.185 (1855)**
1G21	MG-ATP-bound structure of a nitrogenase protein complex between leu127del-Fe protein and the MoFe protein	0.439 (1908)	5VQ4	Nitrogenase Av1 at pH 5	0.253 (1876)
1L5H	FeMo-cofactor Deficient Nitrogenase MoFe Protein	0.400 (792) one NifDK in AU	6BBL	Crystal structure of the a-96Gln MoFe protein variant in the presence of the substrate acetylene	0.221 (1862)
1M1N	Nitrogenase MoFe protein from *Azotobacter vinelandii*	0.288 (1916)	6CDK	Characterization of the P1+ intermediate state of nitrogenase P-cluster	0.243 (1858)
1M1Y	Chemical Crosslink of Nitrogenase MoFe Protein and Fe Protein	0.304 (1908)	6O7L	Nitrogenase MoFeP mutant S188A from *Azotobacter vinelandii* in the dithionite reduced state after redox cycling	0.223 (1849)
1M34	Nitrogenase Complex From *Azotobacter vinelandii* Stabilized By ADP-Tetrafluoroaluminate	0.283 (1912)	6O7M	Nitrogenase MoFeP mutant F99Y from *Azotobacter vinelandii* in the indigo carmine oxidized state	0.235 (1832)
1N2C	Nitrogenase complex from *Azotobacter vinelandii* stabilized by ADP-tetrafluoroaluminate	0.369 (1912)	6O7N	Nitrogenase MoFeP mutant F99Y/S188A from *Azotobacter vinelandii* in the indigo carmine oxidized state	0.220 (1811)
**2AFH**	Crystal Structure of Nucleotide-Free Av2-Av1 Complex	**0.204 (1867)**	6O7O	Nitrogenase MoFeP mutant F99Y/S188A from *Azotobacter vinelandii* in the dithionite reduced state after redox cycling	0.216 (1840)
2AFI	Crystal Structure of MgADP bound Av2-Av1 Complex	0.280 (1889)	6O7P	Nitrogenase MoFeP mutant F99Y from *Azotobacter vinelandii* in the dithionite reduced state	0.224 (1839)
2MIN	Nitrogenase mofe protein from *Azotobacter vinelandii*, oxidized state	0.222 (1885)	6O7Q	Nitrogenase MoFeP mutant S188A from *Azotobacter vinelandii* in the dithionite reduced state after redox cycling	0.215 (1846)
3K1A	Insights into substrate binding at FeMo-cofactor in nitrogenase from the structure of an alpha-70Ile MoFe protein variant	0.235 (1859)	6O7R	Nitrogenase MoFeP mutant F99Y, S188A from *Azotobacter vinelandii* in the dithionite reduced state	0.237 (1871)
3MIN	Nitrogenase MoFe protein from *Azotobacter vinelandii*, oxidized state	0.238 (1865)	**6O7S**	Nitrogenase MoFeP mutant S188A from *Azotobacter vinelandii* in the indigo carmine oxidized state	**0.206 (1842)**
3U7Q	*A. vinelandii* nitrogenase MoFe protein at atomic resolution	0.270 (1887)	6OP1	Selenium incorporated, carbon monoxide inhibited FeMo-cofactor of *Azotobacter vinelandii*	0.226 (1839)
4ND8	Av Nitrogenase MoFe Protein High pH Form	0.239 (1817)	6OP2	Selenium incorporated FeMo-cofactor of nitrogenase from *Azotobacter vinelandii* at high concentration of selenium	0.269 (1876)
4TKU	Reactivated Nitrogenase MoFe-protein from *A. vinelandii*	0.267 (1867)	6OP3	Selenium incorporated FeMo-cofactor of nitrogenase from *Azotobacter vinelandii* with low concentration of selenium	0.271 (1869)
4TKV	CO-bound Nitrogenase MoFe-protein from *A. vinelandii*	0.251 (1874)	6OP4	Selenium-incorporated, carbon monoxide-inhibited, reactivated FeMo-cofactor of nitrogenase from *Azotobacter vinelandii*	0.301 (1915)
4WNA	Structure of the Nitrogenase MoFe Protein from *Azotobacter vinelandii* Pressurized with Xenon	0.243 (1860)	6UG0	N2-bound Nitrogenase MoFe-protein from *Azotobacter vinelandii*	0.232 (1920)
**4WZA**	Asymmetric Nucleotide Binding in the Nitrogenase Complex	**0.203 (1850)**	6VXT	Activated Nitrogenase MoFe-protein from *Azotobacter vinelandii*	0.205 (1910)
4WZB	Crystal Structure of MgAMPPCP-bound Av2-Av1 complex	0.234 (1887)	7JRF	CO-CO-bound nitrogenase MoFe-protein from *A. vinelandii*	0.250 (1816)
4XPI	Fe protein independent substrate reduction by nitrogenase variants altered in intramolecular electron transfer	0.248 (1919)	5BVG	Selenium incorporated nitrogenase MoFe-protein (Av1-Se2B) from *A. vinelandii*	0.255 (1874)

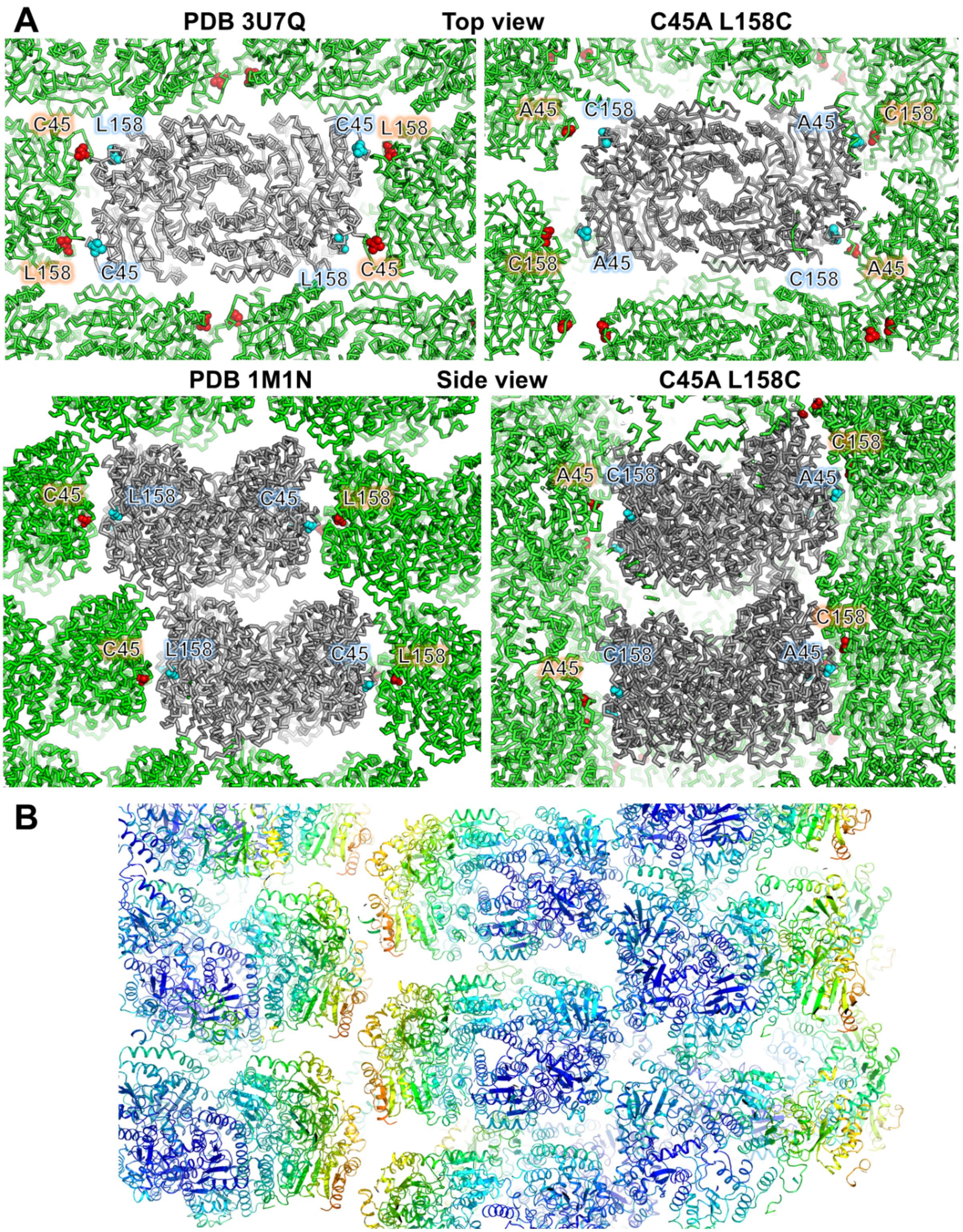

Figure S2: Influence of the mutations on the crystal packing. (**A)** The Cα backbone of the protein is represented as a ribbon. Residues at positions 45 and 158 on the α-subunit are shown as balls. The two nitrogenases of the asymmetric unit are colored in grey, while the nitrogenases constituting the crystal packing are colored in green. (**B)** Crystal packing of C45A L158C double mutant. The anisotropy is illustrated by the B-factor color code from blue (low values) to red (high values).

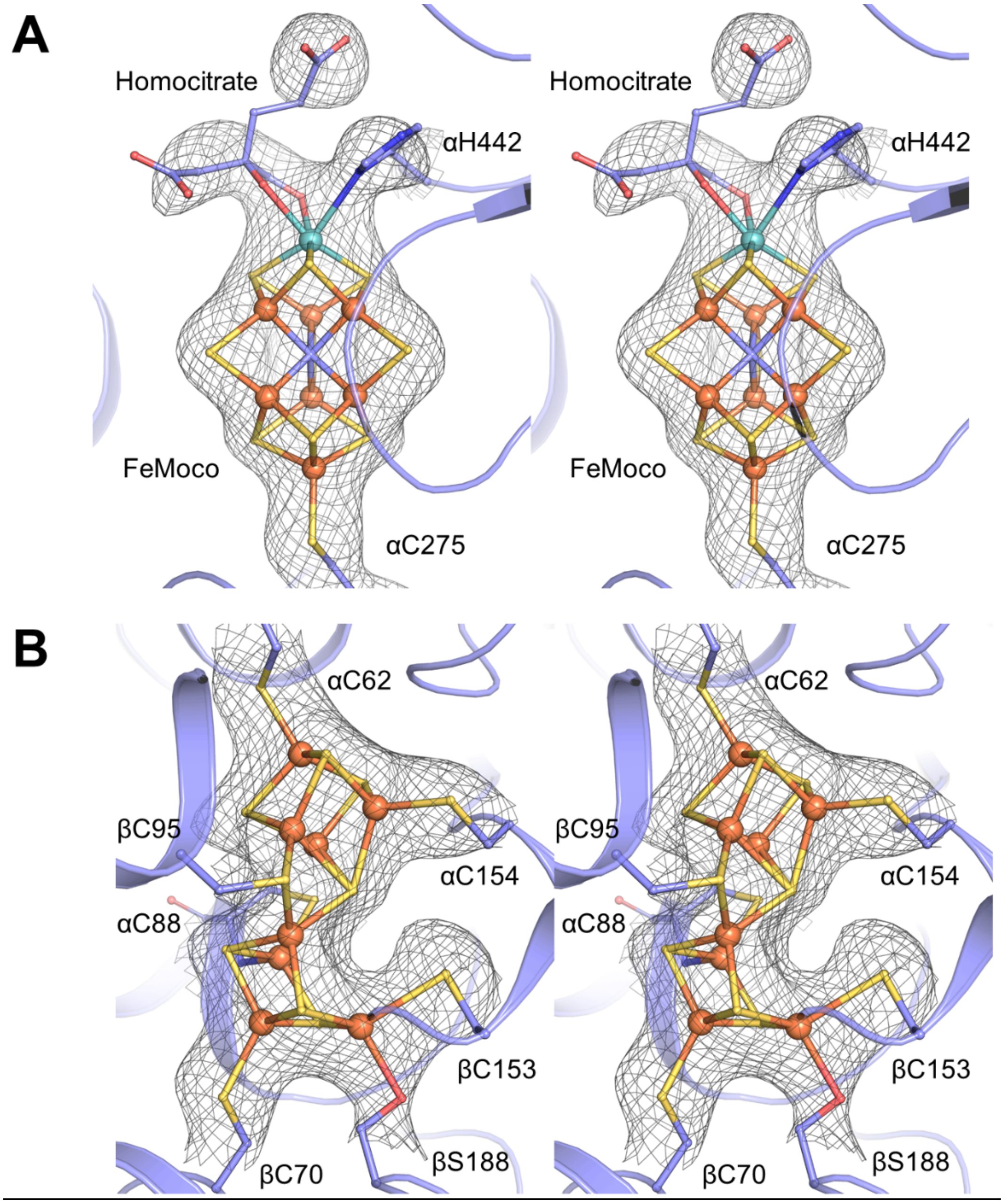

Figure S3: Integrity of the FeMo cofactors and P clusters in the crystallized α-C45A/L158C MoFe proteins. Stereo view of the FeMoco (**A**) and P cluster (**B**). The 2F_o-F_c map, contoured at 1.5 σ is shown in black mesh and the residues coordinating the clusters are in sticks.

Synthesis of the maleimide-desthiobiotin inhibitor ("DTB inhibitor")

General Information: Anhydrous conditions were set up under an inert atmosphere (Ar) utilizing glassware that were oven dried and cooled under Ar purging or under N_2 using standard Schlenk line apparatus. Starting materials were purchased directly from commercial suppliers (Sigma Aldrich, Acros, Alfa Aesar, Fluorochem) and used without further purification unless otherwise stated. All solvents were dried according to standard procedures or bought from commercial suppliers. Reactions were monitored by LC-MS (DIONEX Ultimate 3000 UHPLC with a Thermo LCQ Fleet Mass Spectrometer System using PINNACLE DB C18 column (1.9 µm, 50 x 2.1 mm) with Thermo Xcalibur 2.2.SP1.48 software and analysis with Thermo Xcalibur Qual Browser 2.2.Sp1.48). Reverse phase chromatography on SNAP Ultra 4.5g C18 column using **Biotage®** Isolera™ Four, **15 mL/min**.
NMR characterisation data (^{1}H NMR, ^{13}C NMR and 2D spectra) were collected at 300 K on a Bruker DRX400 (400 MHz) using $CDCl_3$ as solvent. Data for ^{1}H NMR are reported as follows: chemical shift (δ ppm), multiplicity (s = singlet, d = doublet, t = triplet, q = quartet, m = multiplet, br = broad), coupling constant (Hz), integration with the solvent resonance as internal standard ($CDCl_3$: δ = 7.26 ppm for ^{1}H, δ = 77.16 ppm for ^{13}C).
For the characterization of new compounds HR ESI-MS was performed on a Xevo G2-S Tof (Waters) and are reported as mass per charge ratio (m/z) calculated and observed.

N-(15-(2,5-dioxo-2,5-dihydro-1H-pyrrol-1-yl)-12-oxo-3,6,9-trioxa-13-azapentadecyl)-6-((4S,5S)-5-methyl-2-oxoimidazolidin-4-yl)hexanamide (maleimide-desthiobiotin ("DTB") inhibitor)

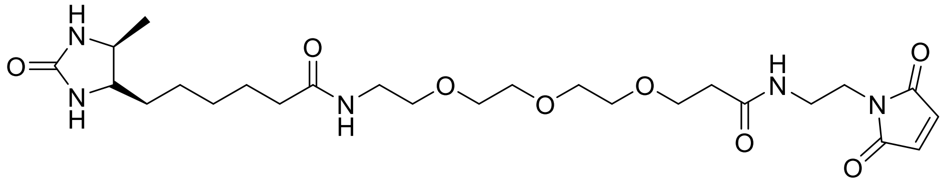

Figure S4: Chemical structure of the DTB inhibitor.

To a solution of 1-(2-aminoethyl) maleimide hydrochloride (13 mg, 0.07 mmol, 1.2 eq.) in dry DMF (1.5 mL), 2,5-dioxopyrrolidin-1-yl 19-((4S,5S)-5-methyl-2-oxoimidazolidin-4-yl)-14-oxo-4,7,10-trioxa-13-azanonadecanoate (31 mg, 0.06 mmol, 1 eq.) and DIPEA (8 mg, 11 µL, 0.06 mmol, 1 eq.) were added at 0 °C. The reaction mixture was stirred at room temperature, under argon, for 15 minutes before the solvent was removed under reduced pressure. The crude reaction mixture was purified by RP flash chromatography (linear gradient 0 – 60% CH_3CN in H_2O with 0.1% TFA) to yield the title compound as a yellow oil (14 mg, 0.025 mmol, 42%). ^{1}H NMR (400 MHz, $CDCl_3$) δ 6.92 (s, 1H), 6.71 (s, 2H), 6.40 (s, 1H), 5.59 (s, 1H), 4.98 (s, 1H), 3.95 – 3.83 (m, 1H), 3.77 – 3.72 (m, 1H), 3.71 – 3.68 (m, 2H), 3.68 – 3.66 (m, 2H), 3.65 – 3.59 (m, 8H), 3.56 (t, *J* = 5.1 Hz, 2H), 3.49 – 3.39 (m, 4H), 2.43 (t, *J* = 5.8 Hz, 2H), 2.20 (t, *J* = 7.4 Hz, 2H), 1.72 – 1.60 (m, 2H), 1.54 – 1.44 (m, 2H), 1.43 – 1.23 (m, 4H), 1.15 (d, *J* = 6.4 Hz, 3H). ^{13}C NMR (101 MHz, $CDCl_3$) δ 173.35, 172.21, 171.08, 163.95, 134.34, 70.48, 70.29, 70.21, 70.16, 70.12, 67.15, 56.30, 51.77, 39.32, 38.46, 37.80, 36.78, 36.18, 29.52, 28.88, 26.02, 25.41, 15.83. LC/MS analysis (linear gradient 30→90% ACN) t_R: 1.54 min, ESI-MS (m/z): $[M+H]^+$: 540.25. HRMS (ESI): calc. for $C_{25}H_{41}N_5O_8$ $[M+Na]^+$ 562.2853, found 562.2867.

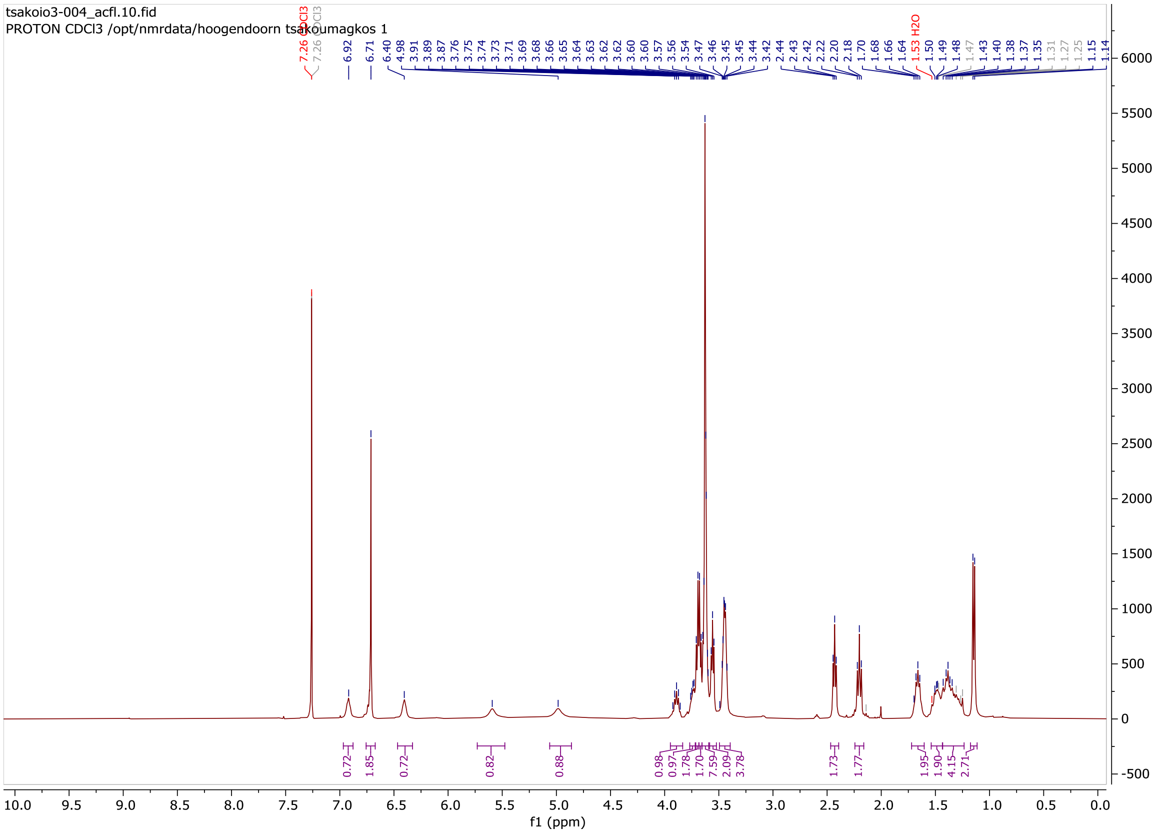

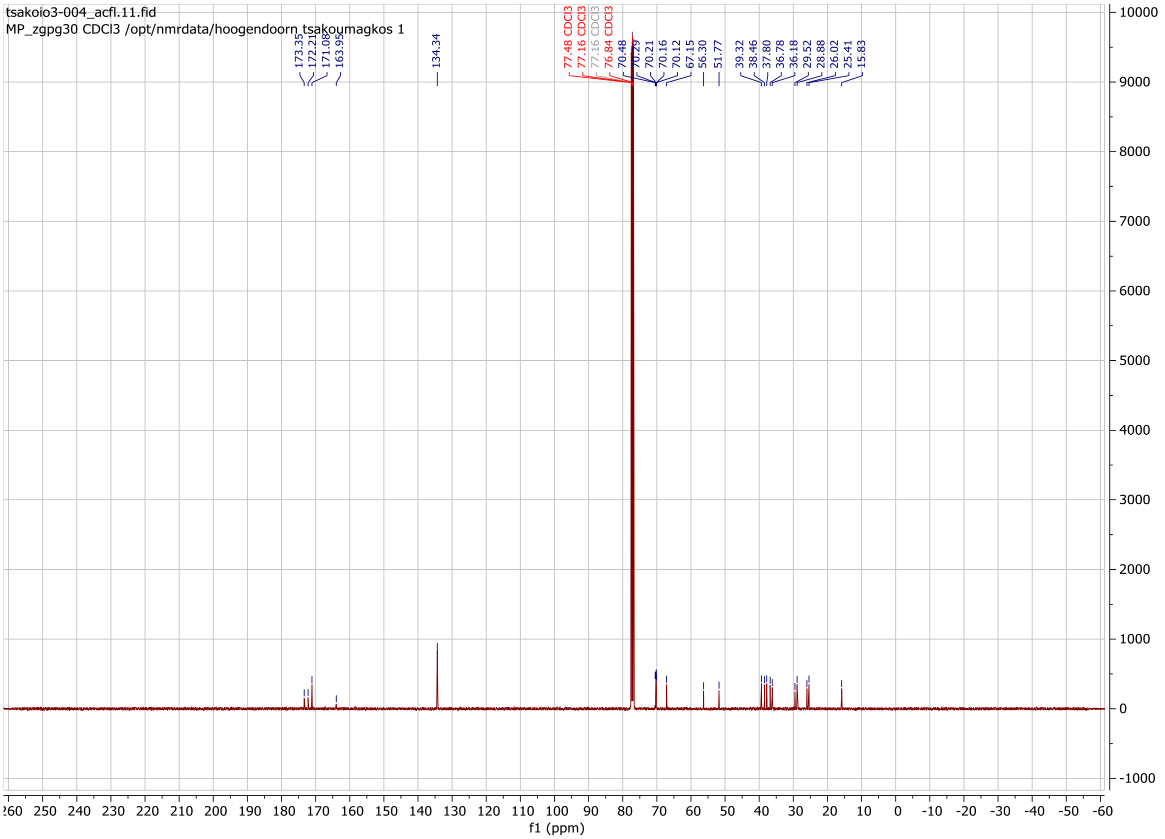

Figure S5: ^{1}H NMR (400 MHz) ^{13}C NMR (101 MHz) spectra of the DTB inhibitor ($CDCl_3$).

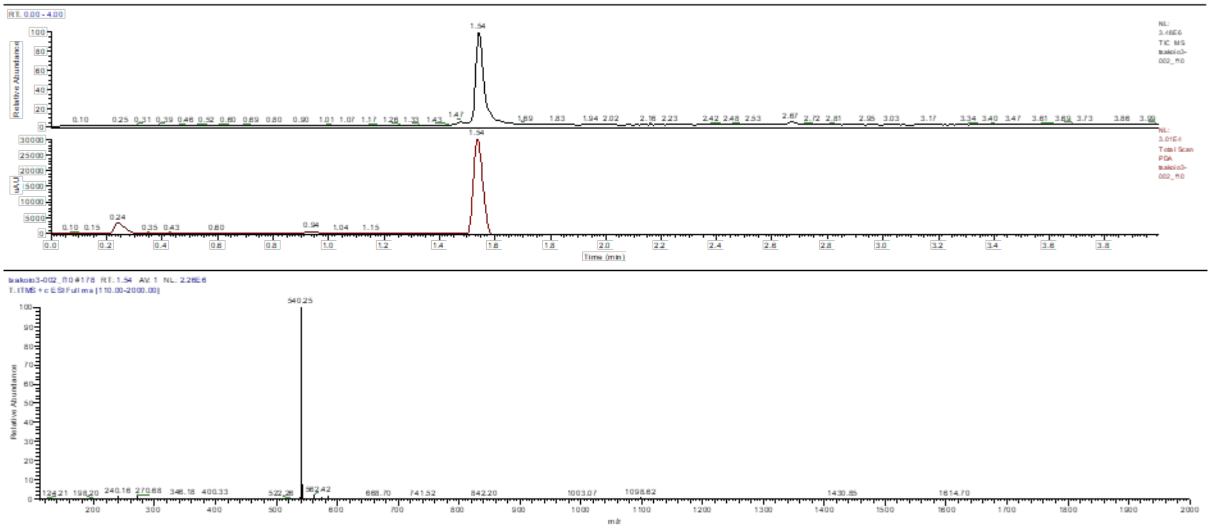

Figure S6: LC-MS of the title compound.

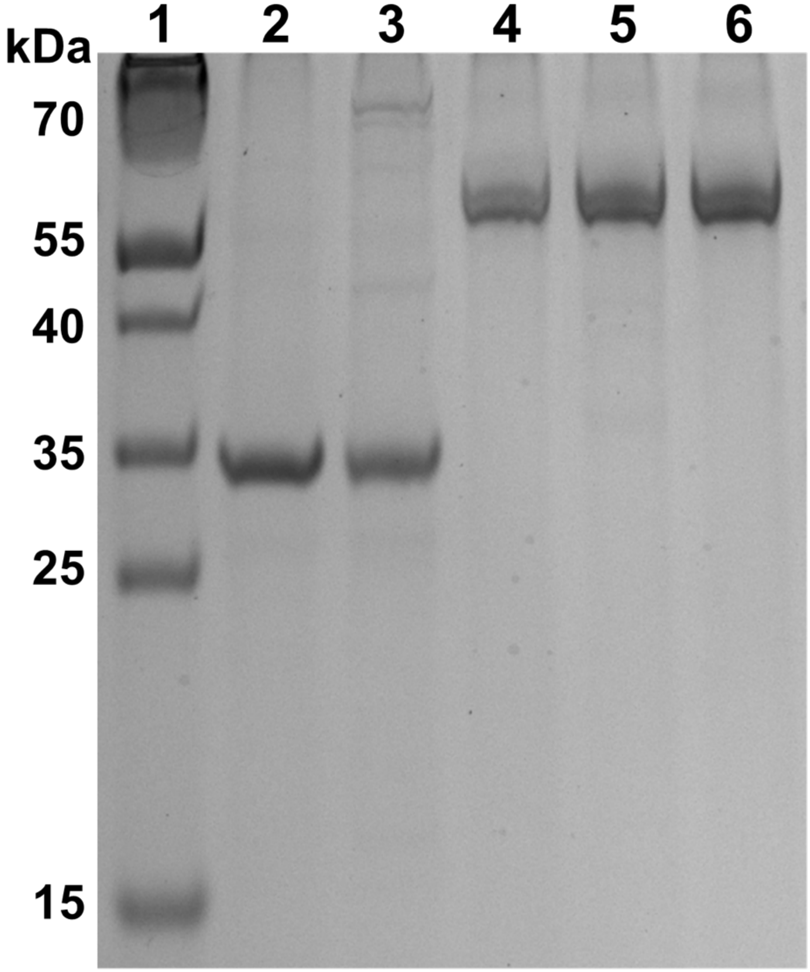

Figure S7: SDS-PAGE (12%), 200 V, Coomassie blue stained. Lanes: 1 = molecular weight markers, 2 = L127Δ Fe protein, 3 = WT Fe protein, 4 = α-C45A/L158C MoFe protein, 5 = WT MoFe protein, 6 = Strep-tagged MoFe protein (N_{term} Strep-II tag on NifD). 400 ng of protein per well.

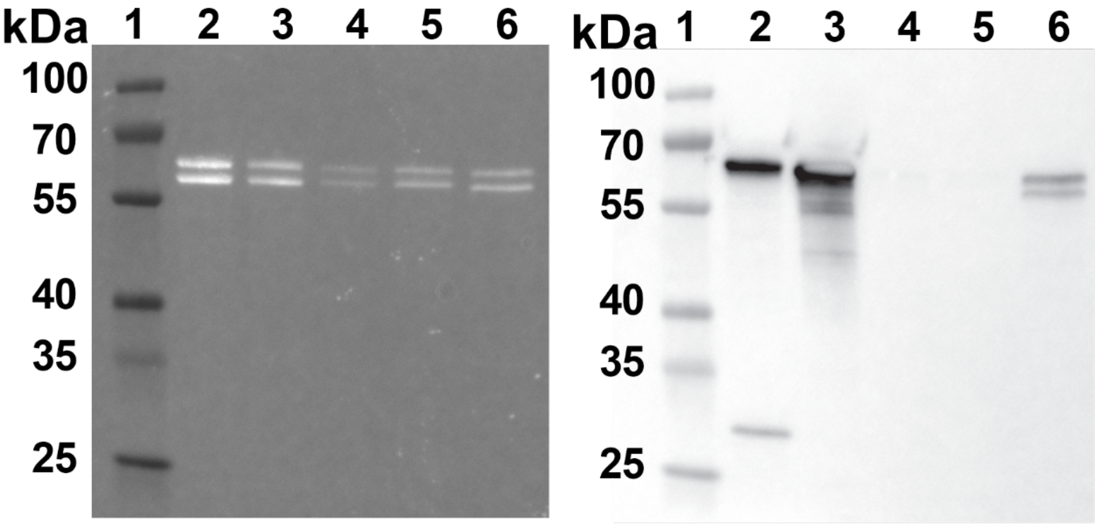

Figure S8: SDS-PAGE analysis (left, strain-free total protein imaging of the PVDF membrane, BioRad) and western blotting with Streptavidin-HRP on WT MoFe protein (right): Lanes: 1 = molecular weight marker; 2 = Strep-tagged WT MoFe protein (N_{term} Strep-II tag on NifD, from *A. vinelandii* strain DJ2102, shared by Dennis Dean – Virginia Tech); 3 = WT MoFe protein treated with DTB inhibitor (DT-free); 4 = "3" although the reaction was performed in the presence of 1 mM DT, 5 = "3" although the reaction was performed in the presence of 10 mM DT, 6 = "3" although the reaction was performed in the presence of 1 mM TCEP. 1 μg of protein per well.

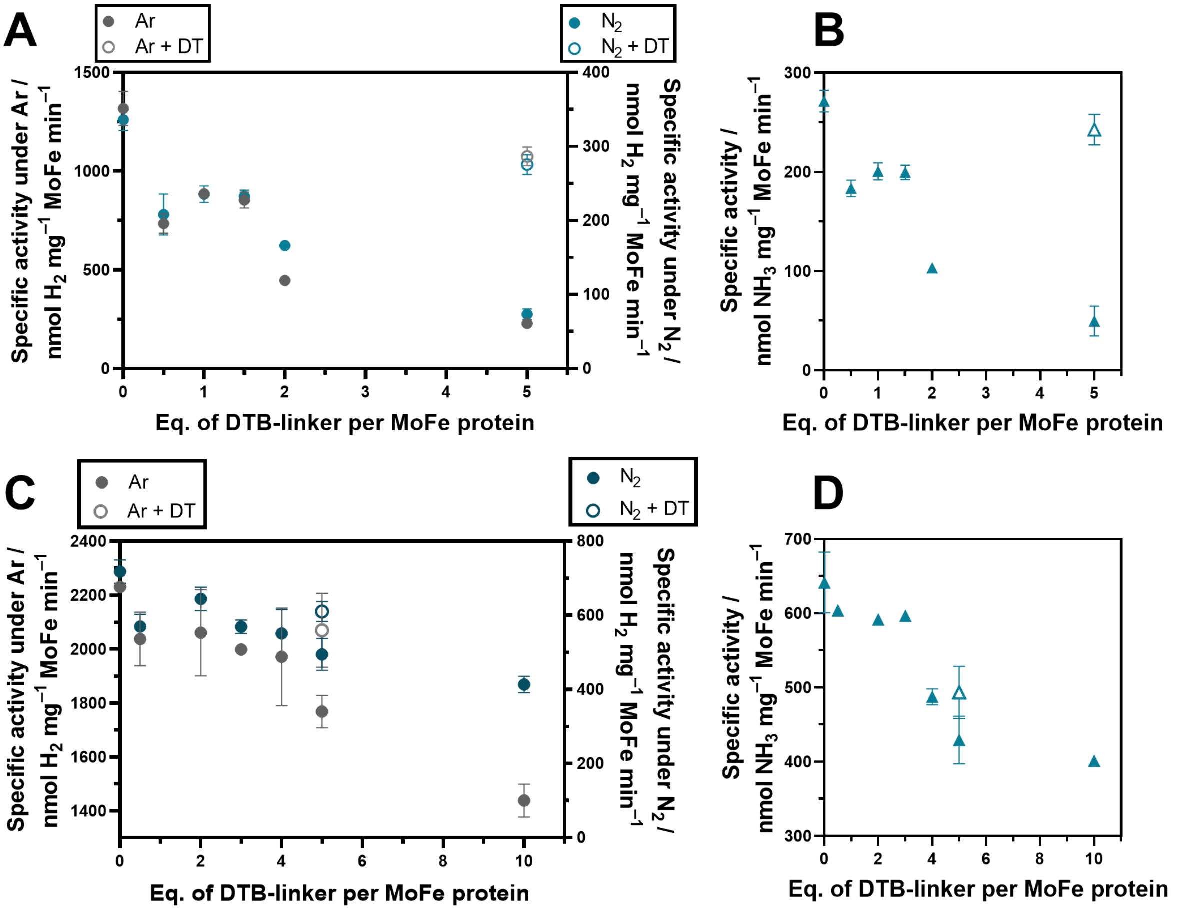

Figure S9: Specific activities of the α-C45A/L158C (top) and WT (bottom) MoFe proteins upon treatment with increasing equivalents of DTB inhibitor (per MoFe protein). **(A)** Specific activity for H_2 evolution, labelled as black dots on the left y axis (under 1 atm Ar) and as blue dots on the right y axis (under 1 atm N_2). Empty dots represent the specific activities obtained when treating the α-C45A/L158C MoFe protein with 10 mM DT before the addition of the DTB inhibitor (to reduce the maleimide). **(B)** Specific activity for NH_3 evolution, labeled as blue triangles under 1 atm N_2 and an empty triangle for α-C45A/L158C MoFe treated with 10 mM DT prior to DTB inhibitor addition. **(C)** Specific activity for H_2 evolution, labelled as black dots on the left y axis (under 1 atm Ar) and as blue dots on the right y axis (under 1 atm N_2). Empty dots represent the specific activities obtained when treating the WT MoFe protein with 10 mM DT before the addition of the DTB inhibitor (to reduce the maleimide and inhibit the Cys conjugation reaction). **(D)** Specific activity for NH_3 evolution, labeled as blue triangles under 1 atm N_2 and an empty triangle for WT MoFe treated with 10 mM DT prior to DTB inhibitor addition. In all cases *n* = 3.

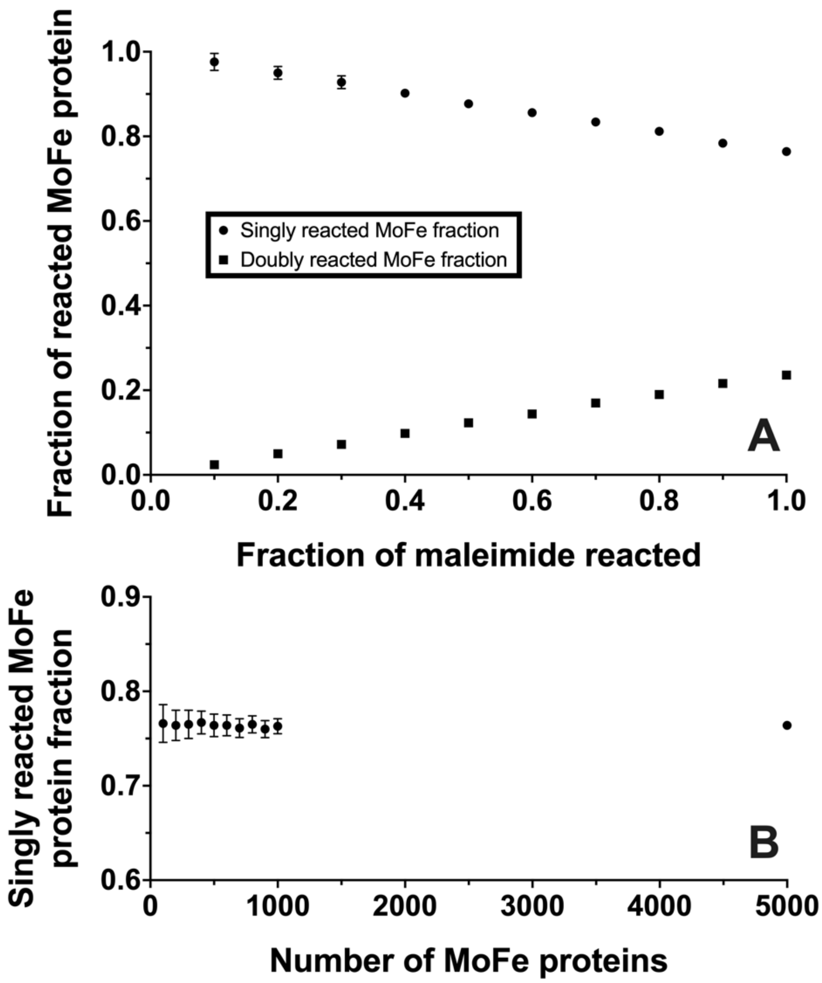

Figure S10: Simulation of singly and doubly reacted α-C45A/L158C MoFe protein fraction upon treatment with 0.5 molar equivalents of maleimide-Streptag inhibitor. (**A**) Simulation of 1000 proteins reacting with 500 maleimide-Streptags (100 simulations for each point, mean and SD reported), represented the fractions of "reacted" MoFe protein that contain singly and doubly reacted MoFe (where a singly reacted MoFe = half-inhibited MoFe). Assumptions: (i) a reaction takes place each time a maleimide-Streptag inhibitor reaches the α-158C position, (ii) no reaction occurs if the doubly reacted α-C45A/L158C MoFe is encountered, (iii) the maleimide-Streptag reaction is completely selective for the α-C158 residue, and (iv) the maleimide is not hydrolyzed or reduced prior to reaching the α-C158 residue. (**B**) Fraction of singly reacted α-C45A/L158C MoFe protein upon scaling up of the simulation at the same ratio of molar equivalents.

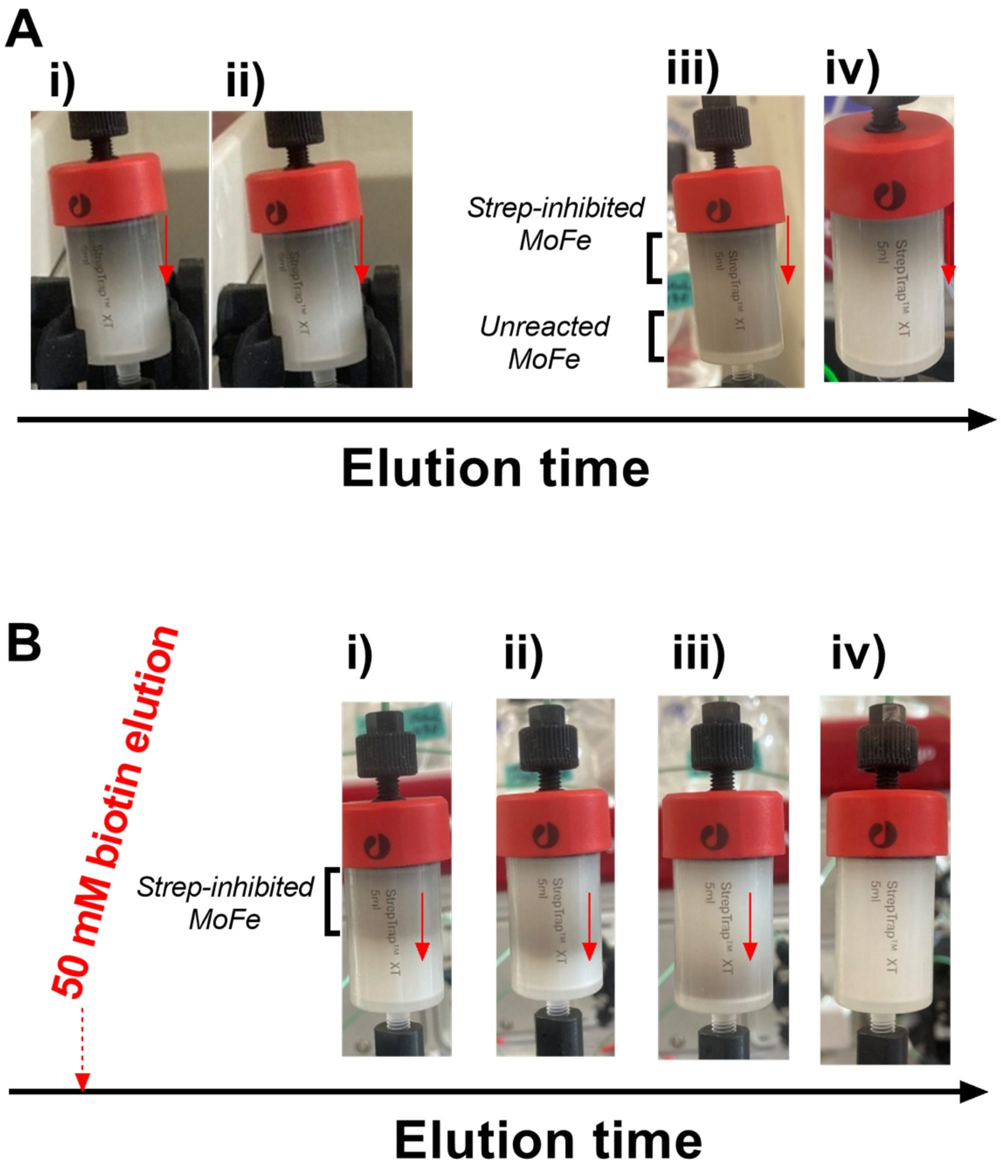

Figure S11: Purification of Strep-inhibited MoFe protein after reaction with the Strep-peptide inhibitor. (**A**) Pictures of the StrepTrap XT column after sample loading (sample contains untreated and Strep-inhibited α-C45A/L158C MoFe protein). Red arrow indicates the direction of elution, and (iii) represents the differentiation between Strep-inhibited MoFe protein binding to the column and unreacted MoFe protein passing as the column flow-through. (**B**) Pictures of the column after elution of the Strep-inhibited MoFe protein with buffer containing 50 mM biotin.

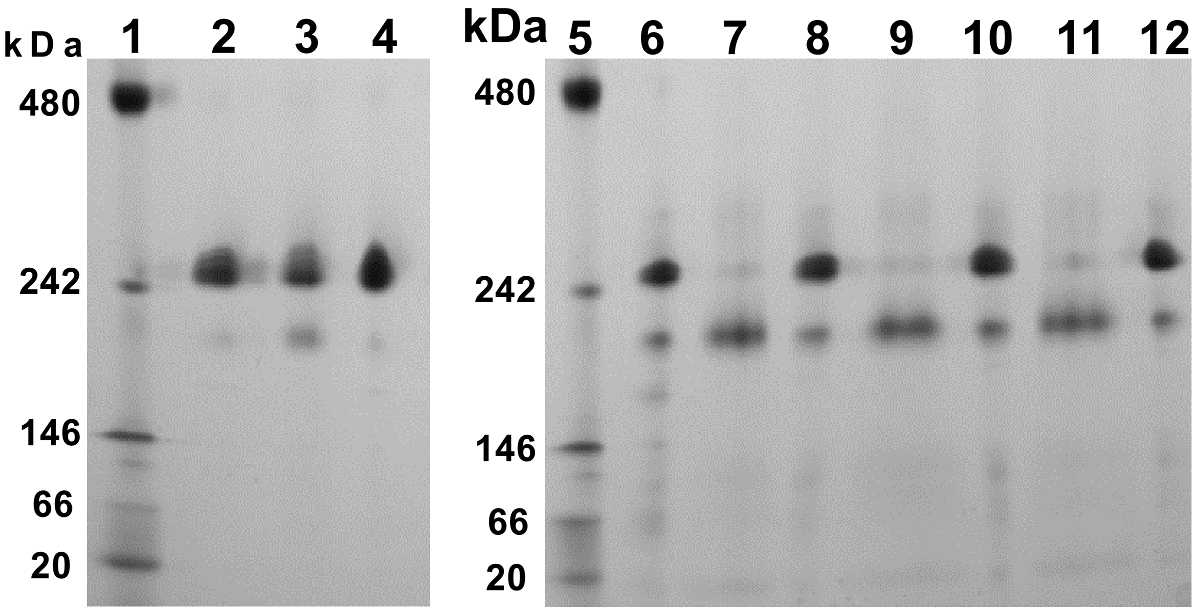

Figure S12: Native PAGE 3-12% acrylamide gradient, anoxic atmosphere (>95% N_2 and <5% H_2), 150 V. Lanes: 1 and 5 = molecular weight markers, 2 = untreated WT MoFe protein, 3 = Strep-inhibited WT MoFe protein (eluted from the StrepTactin XT solid phase), 4= unmodified WT MoFe protein that did not tightly bind to the column solid phase (unreacted flow-through), 6 = unreacted α-C45A/L158C MoFe protein, 7/9/11 = Strep-inhibited α-C45A/L158C MoFe protein (eluted from the StrepTactin XT solid phase); 8/10/12 = unmodified α-C45A/L158C MoFe protein that did not tightly bind to the column solid phase (unreacted flow-through). 7/8, 9/10 and 11/12 represent three independent α-C45A/L158C MoFe protein functionalization reactions. Strep-inhibited wild-type MoFe protein was prepared as detailed for the α-C45A/L158C MoFe protein. 1.5 μg protein per well.

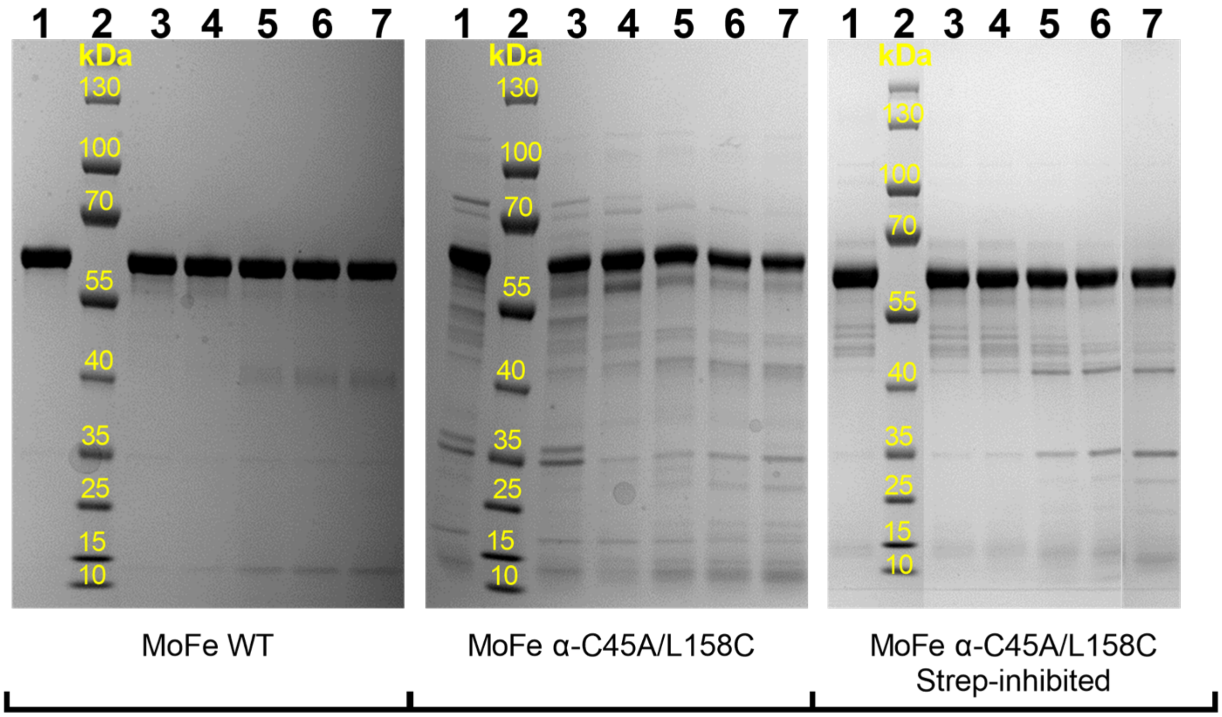

Figure S13: SDS-PAGE analysis for temperature-controlled limited proteolysis (Coomassie blue staining). Lanes: 1 = MoFe protein at t = 0 min after trypsin addition, 2 = molecular weight markers, 3 = MoFe protein at t = 2 min, 4 = MoFe protein at t = 5 min, 5 = MoFe protein at t = 30 min, 6 = MoFe protein at t = 60 min, 7 = MoFe protein at t = 90 min. *Note*: Lane 7 in the image of the SDS-PAGE gel for the Strep-inhibited α-C45A/L158C MoFe protein was cut and reshuffled after analysis, in order to clearly present the time-course of the experiment (due to the sample loading sequence on the gel). 1.5-1.75 μg of protein per well.

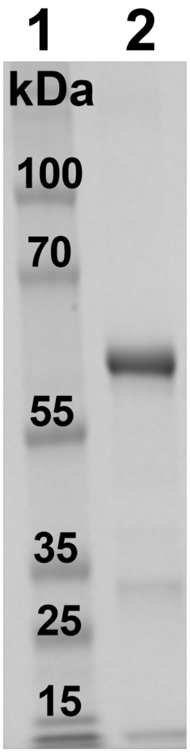

Figure S14: SDS-PAGE analysis of Strep-inhibited α-C45A/L158C MoFe protein was incubated with 5 molar equivalents of L127Δ Fe protein further purified by His Trap column. Lanes: 1 = molecular weight markers, 2 = Strep-inhibited α-C45A/L158C MoFe protein was incubated with 5 molar equivalents of L127Δ Fe protein further purified by His Trap column. 2 μg of protein per well.

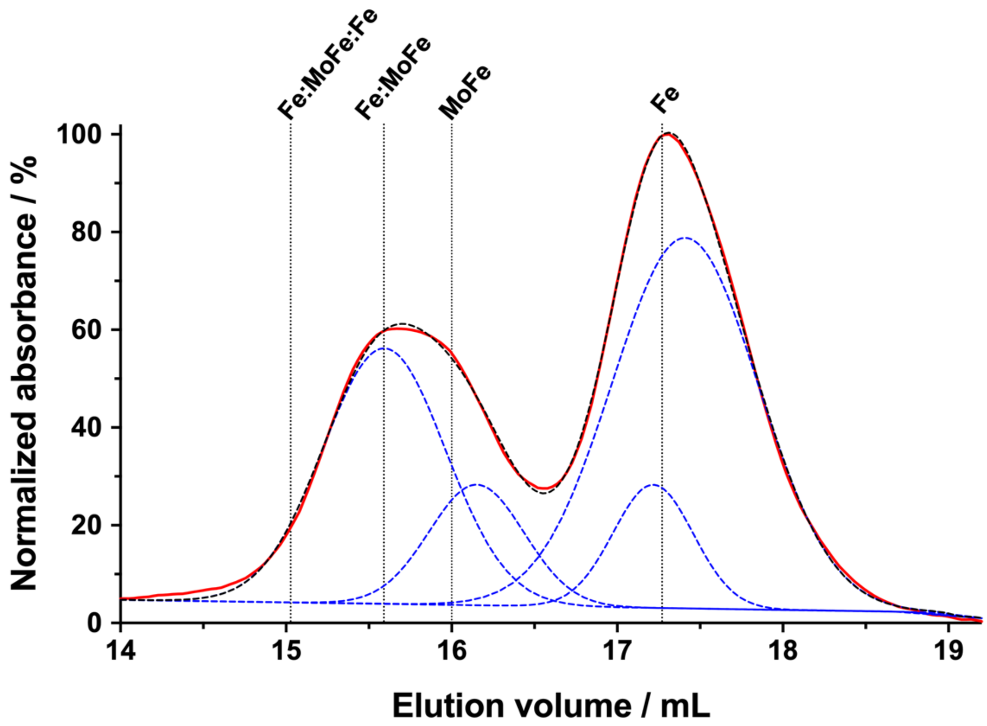

Fitted Peak / mL	Max. Peak Height (%)
15.59	52.3
16.15	24.7
17.22	25.2
17.41	75.8

Figure S15: Multi-Gaussian peak fitting (performed using Origin Pro) of the gel filtration profile obtained for Strep-reacted α-C45A/L158C MoFe protein (half-inhibited MoFe protein targeted, as detailed in the experimental section) after incubation with 5 molar equivalents of L127Δ Fe protein, extracted from Figure 5D in the main article. The experimental profile is shown in red, whereas the individual fitted peaks are shown in blue dashed lines and the cumulative predicted profile is shown by the black dashed line. The vertical lines indicating the elution volumes of the four protein components were extracted from Figure 5D in the main article (the apparent peaks in the red trace).

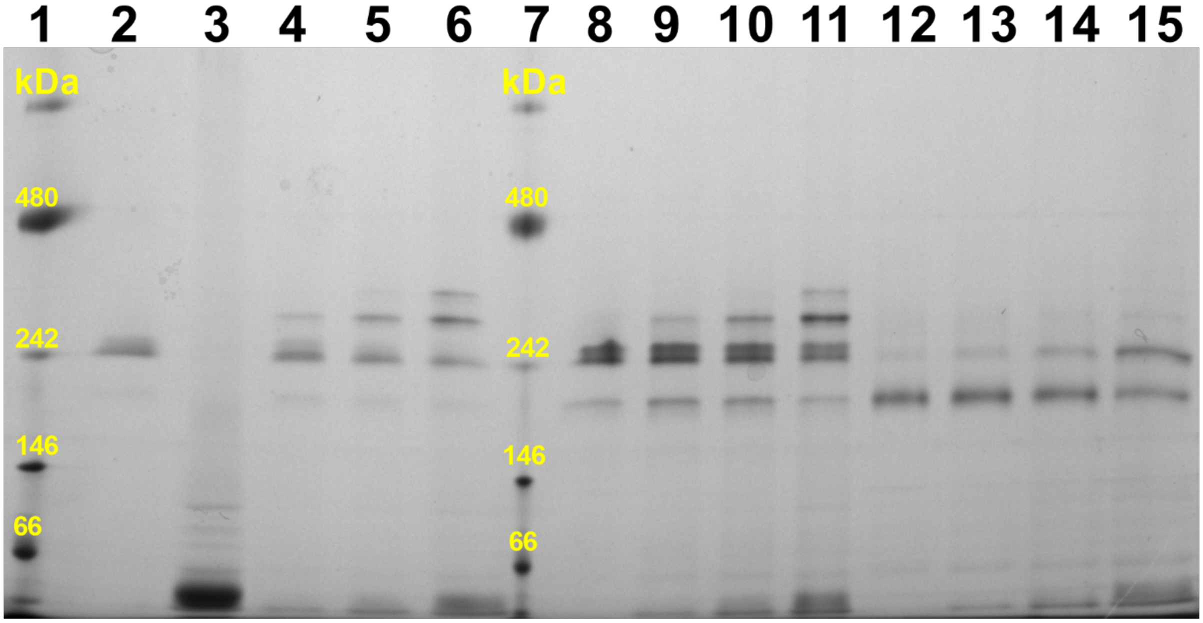

Figure S16: Anoxic Native PAGE 3-12%, 150 V. Lanes: 1,7 = molecular weight markers, 2 = WT MoFe protein, 3 = L127Δ Fe protein, 4 = WT MoFe + L127Δ Fe (1:1), 5 = WT MoFe + L127Δ Fe (1:2), 6 = WT MoFe + L127Δ Fe (1:5), 8 = α-C45A/L158C MoFe, 9 = α-C45A/L158C MoFe + L127Δ Fe (1:1), 10 = α-C45A/L158C MoFe + L127Δ Fe (1:2), 11 = α-C45A/L158C MoFe + L127Δ Fe (1:5), 12 = Strep-inhibited α-C45A/L158C MoFe, 13 = Strep-inhibited α-C45A/L158C MoFe + L127Δ Fe (1:1), 14 = Strep-inhibited α-C45A/L158C MoFe + L127Δ Fe (1:2), 15 = Strep-inhibited α-C45A/L158C MoFe + L127Δ Fe (1:5).

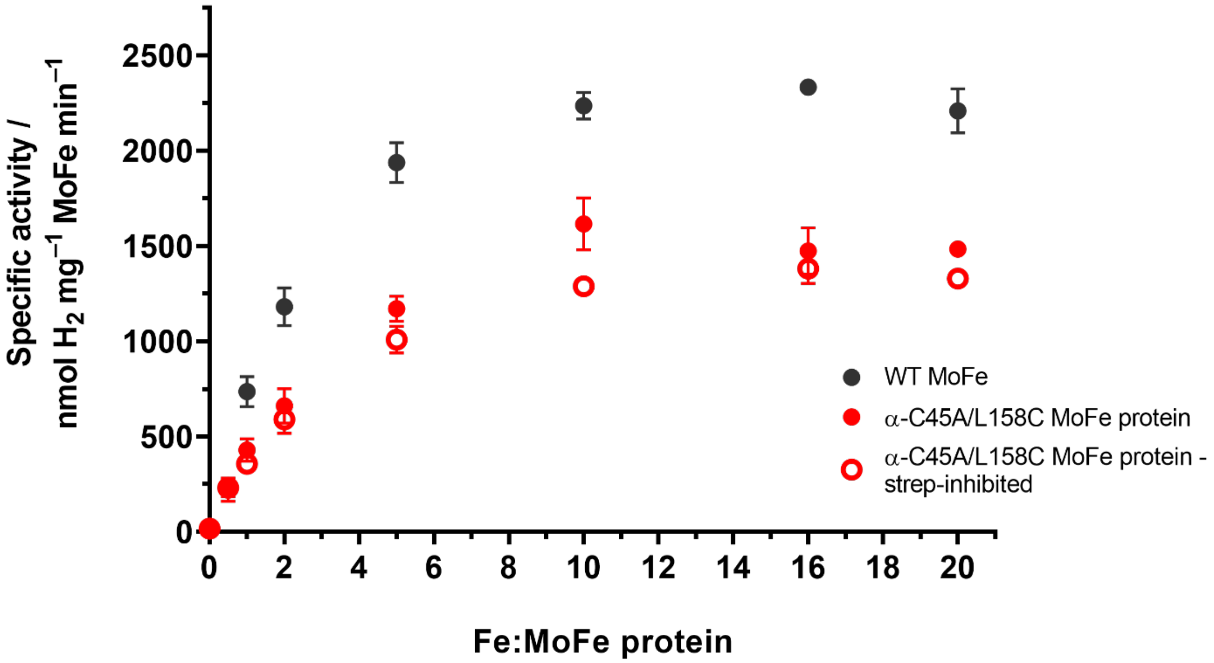

Figure S17: Fe protein titration. Specific activities for H_2 evolution under 1 atm Ar. The x axis indicates the molar equivalents of Fe protein per MoFe protein. Black dots = WT MoFe protein, red filled dots = α-C45A/L158C MoFe and red empty dots = Strep-inhibited α-C45A/L158C MoFe. *n* = 3, error bars report standard deviation. All assays contained 0.1 mg mL^{-1} MoFe protein and were performed for 8 minutes at 30 °C. Note = the specific activities of the α-C45A/L158C MoFe proteins (native and Strep-inhibited) were performed using protein isolated from a large-scale *A. vinelandii* culture (single biological repeat).

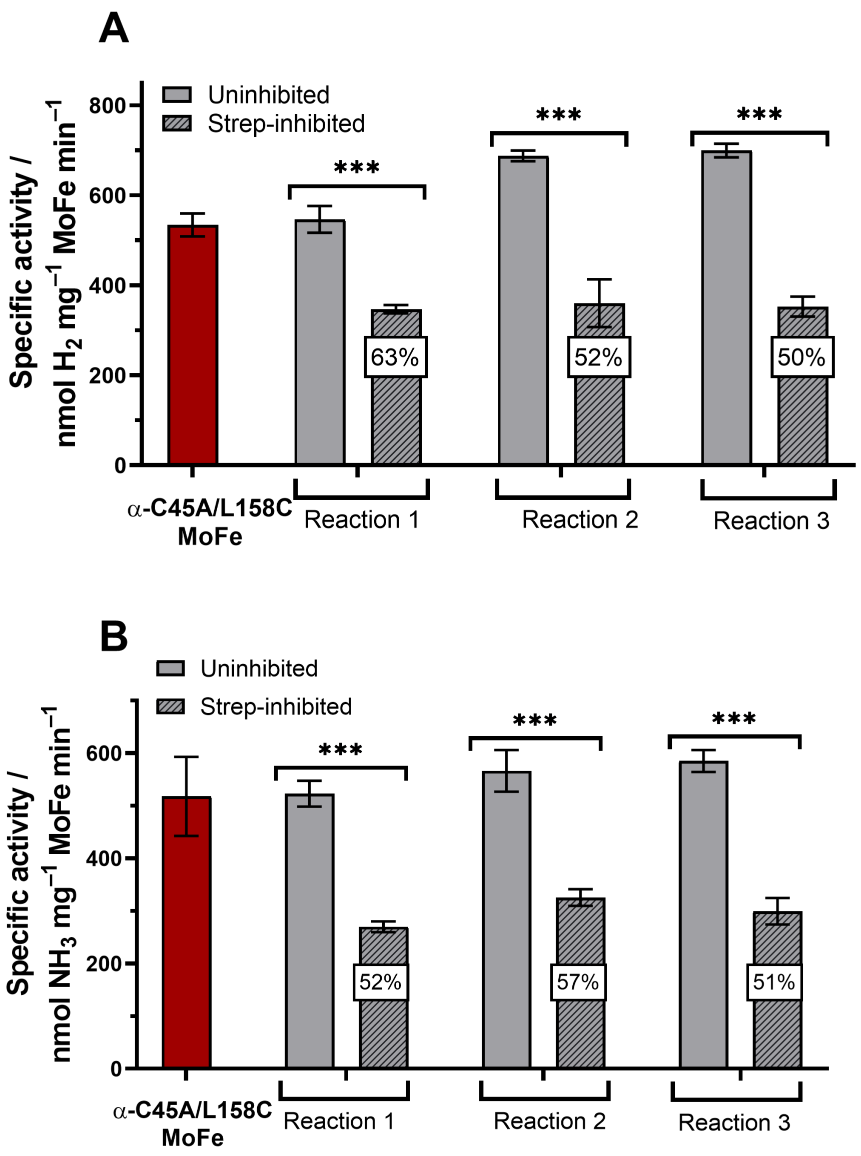

Figure S18: (**A**) Specific activity for H_2 evolution under 1 atm N_2 atmosphere. Red is the standard α-C45A/L158C MoFe purified in the absence of DT. Light grey represents the flow-through (uninhibited) MoFe proteins from three functionalization reactions, grey cross-hatched bars represent the Strep-inhibited α-C45A/L158C MoFe. Percentage value in white square corresponds to the remaining specific activity of each Strep-inhibited MoFe divided in relation to its unreacted MoFe (flow-through) fraction. (**B**) Specific activity for NH_3 evolution under 1 atm N_2 atmosphere. All activity assays were performed for 8 minutes at 30 °C with 0.1 mg mL^{-1} MoFe protein and 16.6 molar equivalents of Fe protein (0.48 mg). (**A-B**) *n* = 3 and error bars report standard deviation (propagated where necessary).

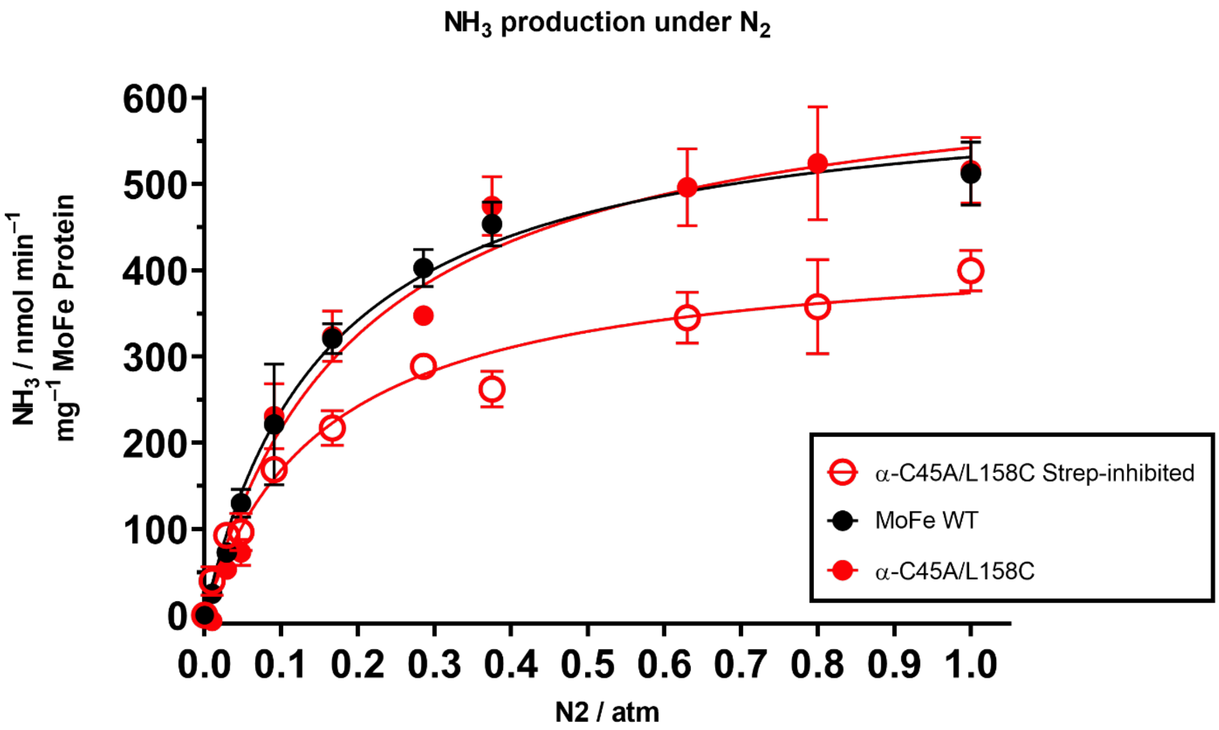

Figure S19: Specific activity of MoFe proteins *vs.* N_2 partial pressure (nmol NH_3 min^{-1} mg^{-1} MoFe). Black data: WT MoFe; red data: uninhibited α-C45A/L158C MoFe; red hollow-circle data: Strep-inhibited α-C45A/L158C MoFe. $n = 3$, error bars report standard deviation. All assays contained 0.1 mg mL^{-1} MoFe protein and were performed for 8 minutes at 30 °C.

Michaelis-Menten parameter	WT MoFe
K_M^{app} (atm)	0.16 ± 0.03
V_{max}^{app} (nmol NH_3 $min^{-1}.mg^{-1}$)	616.8 ± 41.6

Table S5: Michaelis-Menten kinetic parameters for WT MoFe protein.

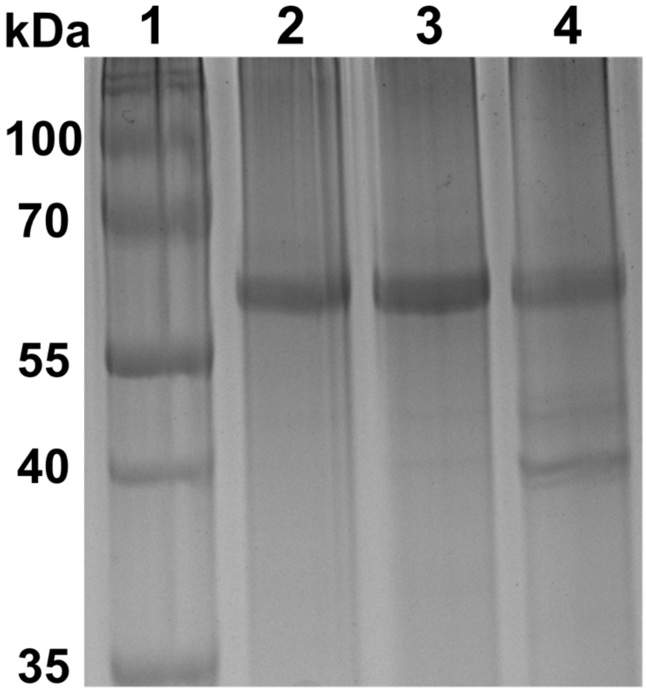

Figure S20: SDS-PAGE (10%, Coomassie blue stained), 180 V. Lanes: 1 = molecular weight markers, 2 = α-C45S MoFe protein, 3 = unreacted α-C45S MoFe protein, 4 = Strep-inhibited α-C45S MoFe. 400 ng of protein per well.

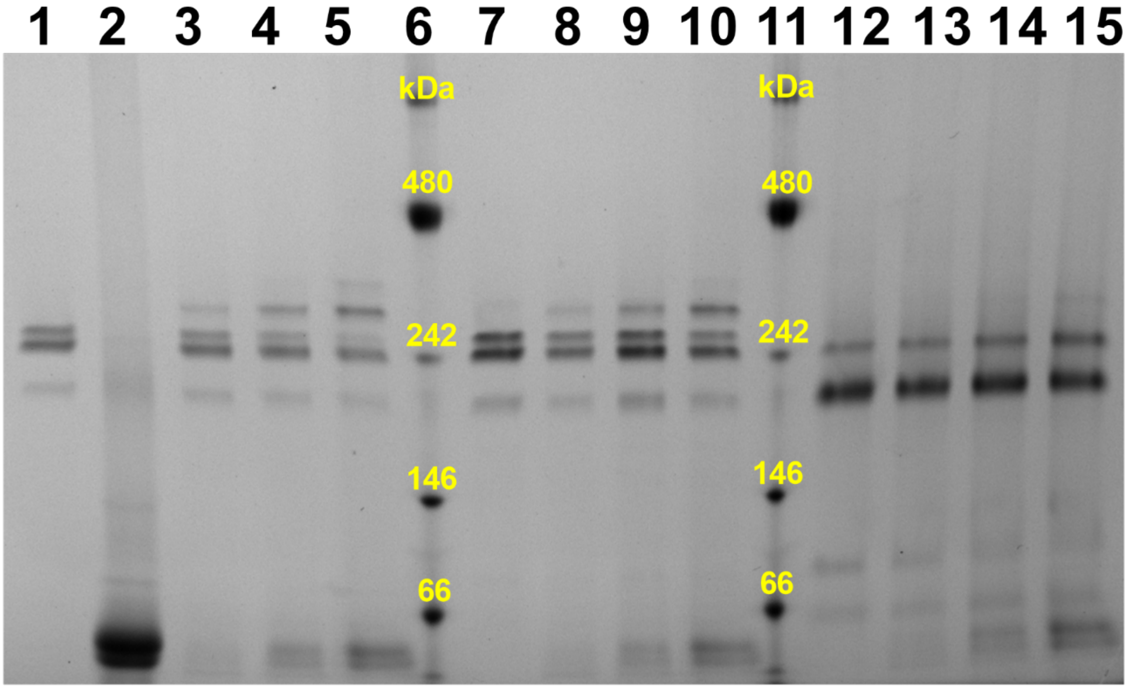

Figure S21: Anoxic Native PAGE 3-12%, 150 V. Lanes: 1 = WT MoFe protein, 2 = L127Δ Fe protein, 3 = WT MoFe + L127Δ Fe (1:1), 4 = WT MoFe + L127Δ Fe (1:2), 5 = WT MoFe + L127Δ Fe (1:5), 6,11 = molecular weight markers, 7 = α-C45S MoFe, 8 = α-C45S MoFe + L127Δ Fe (1:1), 9 = α-C45S MoFe + L127Δ Fe (1:2), 10 = α-C45S MoFe + L127Δ Fe (1:5), 12 = Strep-inhibited α-C45S MoFe, 13 = Strep-inhibited α-C45S MoFe + L127Δ Fe (1:1), 14 = Strep-inhibited α-C45S MoFe + L127Δ Fe (1:2), 15 = Strep-inhibited α-C45S MoFe + L127Δ Fe (1:5).

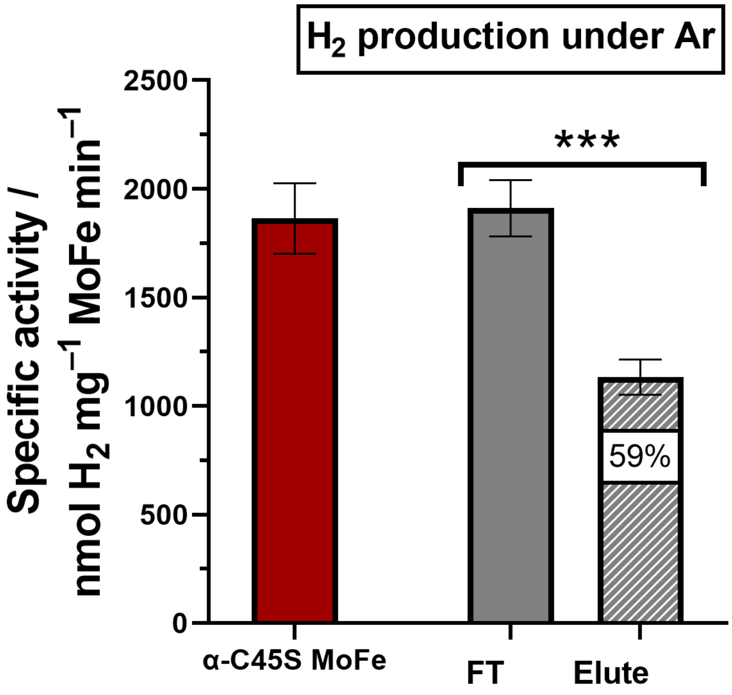

Figure S22: Specific activity for H_2 evolution under 1 atm Ar atmosphere. Red is the standard α-C45S MoFe purified in the absence of DT. Light grey represents the flow-through (uninhibited) MoFe proteins from functionalization reaction, grey cross-hatched bars represent the Strep-inhibited α-C45S MoFe. Percentage value in white square corresponds to the remaining specific activity of Strep-inhibited MoFe divided in relation to its unreacted MoFe (flow-through) fraction. All activity assays were performed for 8 minutes at 30 °C with 0.1 mg mL^{-1} MoFe protein and 16.6 molar equivalents of Fe protein (0.48 mg). n = 3 and error bars report standard deviation (propagated where necessary).

References

(1) Milton, R. D.; Cai, R.; Abdellaoui, S.; Leech, D.; De Lacey, A. L.; Pita, M.; Minteer, S. D. Bioelectrochemical Haber–Bosch Process: An Ammonia-Producing H2/N2 Fuel Cell. *Angew. Chemie - Int. Ed.* **2017**, *56* (10), 2680–2683. https://doi.org/10.1002/anie.201612500.

(2) Roth, L. E.; Tezcan, F. A. ATP-Uncoupled, Six-Electron Photoreduction of Hydrogen Cyanide to Methane by the Molybdenum–Iron Protein. *J. Am. Chem. Soc.* **2012**, *134* (20), 8416–8419. https://doi.org/10.1021/ja303265m.

(3) Puri, A. W.; Owen, S.; Chu, F.; Chavkin, T.; Beck, D. A. C.; Kalyuzhnaya, M. G.; Lidstrom, M. E. Genetic Tools for the Industrially Promising Methanotroph Methylomicrobium Buryatense. *Appl. Environ. Microbiol.* **2015**, *81* (5), 1775–1781. https://doi.org/10.1128/AEM.03795-14.

(4) Gu, W.; Ul Haque, M. F.; Baral, B. S.; Turpin, E. A.; Bandow, N. L.; Kremmer, E.; Flatley, A.; Zischka, H.; DiSpirito, A. A.; Semrau, J. D. A TonB-Dependent Transporter Is Responsible for Methanobactin Uptake by Methylosinus Trichosporium OB3b. *Appl. Environ. Microbiol.* **2016**, *82* (6), 1917–1923. https://doi.org/10.1128/AEM.03884-15.

(5) Schäfer, A.; Tauch, A.; Jäger, W.; Kalinowski, J.; Thierbach, G.; Pühler, A. Small Mobilizable Multi-Purpose Cloning Vectors Derived from the Escherichia Coli Plasmids PK18 and PK19: Selection of Defined Deletions in the Chromosome of Corynebacterium Glutamicum. *Gene* **1994**, *145* (1), 69–73. https://doi.org/10.1016/0378-1119(94)90324-7.

(6) Christiansen, J.; Goodwin, P. J.; Lanzilotta, W. N.; Seefeldt, L. C.; Dean, D. R. Catalytic and Biophysical Properties of a Nitrogenase Apo-MoFe Protein Produced by a NifB-Deletion Mutant of Azotobacter Vinelandii. *Biochemistry* **1998**, *37* (36), 12611–12623. https://doi.org/10.1021/bi981165b.

(7) Ryle, M. J.; Seefeldt, L. C. Elucidation of a MgATP Signal Transduction Pathway in the Nitrogenase Iron Protein: Formation of a Conformation Resembling the MgATP-Bound State by Protein Engineering. *Biochemistry* **1996**, *35* (15), 4766–4775. https://doi.org/10.1021/bi960026w.

(8) Lanzilotta, W. N.; Fisher, K.; Seefeldt, L. C. Evidence for Electron Transfer from the Nitrogenase Iron Protein to the Molybdenum-Iron Protein without MgATP Hydrolysis: Characterization of a Tight Protein-Protein Complex. *Biochemistry* **1996**, *35* (22), 7188–7196. https://doi.org/10.1021/bi9603985.

(9) Jimenez-Vicente, E.; Yang, Z. Y.; Keith Ray, W.; Echavarri-Erasun, C.; Cash, V. L.; Rubio, L. M.; Seefeldt, L. C.; Dean, D. R. Sequential and Differential Interaction of Assembly Factors during Nitrogenase MoFe Protein Maturation. *J. Biol. Chem.* **2018**, *293* (25), 9812–9823. https://doi.org/10.1074/jbc.RA118.002994.

(10) Milton, R. D.; Abdellaoui, S.; Khadka, N.; Dean, D. R.; Leech, D.; Seefeldt, L. C.; Minteer, S. D. Nitrogenase Bioelectrocatalysis: Heterogeneous Ammonia and Hydrogen Production by MoFe Protein. *Energy Environ. Sci.* **2016**, *9* (8), 2550–2554. https://doi.org/10.1039/c6ee01432a.

(11) Baldwin, A. D.; Kiick, K. L. Tunable Degradation of Maleimide-Thiol Adducts in Reducing Environments. *Bioconjug. Chem.* **2011**, *22* (10), 1946–1953. https://doi.org/10.1021/bc200148v.
